# Mechanical power is not associated with mortality in COVID-19 mechanically ventilated patients

**DOI:** 10.1186/s13613-025-01430-6

**Published:** 2025-02-25

**Authors:** Enric Barbeta, Cláudia Barreiros, Edoardo Forin, Amedeo Guzzardella, Anna Motos, Laia Fernández-Barat, Albert Gabarrús, Adrián Ceccato, Ricard Ferrer, Jordi Riera, Oscar Peñuelas, José Ángel Lorente, David de Gonzalo-Calvo, Jessica Gonzalez, Rosario Amaya-Villar, José Manuel Añón, Ana Balan, Carme Barberà, José Barberán, Aaron Blandino, Maria Victoria Boado, Elena Bustamante-Munguira, Jesús Caballero, María Luisa Cantón-Bulnes, Cristina Carbajales, Nieves Carbonell, Mercedes Catalán-González, Nieves Franco, Cristóbal Galbán, Víctor D. Gumucio-Sanguino, Maria Del Carmen de la Torre, Emilio Díaz, Ángel Estella, Elena Gallego, José Manuel Gómez, Arturo Huerta, Ruth Noemí Jorge García, Ana Loza-Vázquez, Judith Marin-Corral, María Cruz Martin Delgado, Amalia Martínez, Ignacio Martínez, Juan Lopez, Guillermo M. Albaiceta, María Teresa Nieto, Mariana Andrea Novo, Yhivian Peñasco, Felipe Pérez-García, Pilar Ricart, Alejandro Rodríguez, Victor Sagredo, Angel Sánchez-Miralles, Susana Sancho, Ferran Roche-Campo, Lorenzo Socias, Jordi Solé-Violan, Luis Tamayo, José Trenado, Alejandro Úbeda, Luis Jorge Valdivia, Pablo Vidal, Ferran Barbé, Jordi Vallverdú, Antoni Torres, CIBERESUCICOVID Project investigators (COV20/00110, ISCIII), Enric Barbeta, Ana Balan, Nieves Franco, Ferran Roche-Campo, Alejandro Úbeda, Berta Adell-Serrano, Alexander Agrifoglio, María Aguilar Cabello, Luciano Aguilera, Victoria Alcaraz-Serrano, Cesar Aldecoa, Cynthia Alegre, Sergio Álvarez, Antonjo Álvarez Ruiz, Rut Andrea, José Ángel, Marta Arrieta, J. Ignacio Ayestarán, Joan Ramon Badia, Mariona Badía, Orville Báez Pravia, Begoña Balsera, Laura Barbena, Tommaso Bardi, Patricia Barral Segade, Marta Barroso, José Ángel Berezo García, Judit Bigas, Rafael Blancas, María Luisa Blasco Cortés, María Boado, María Bodi Saera, Neus Bofill, María Teresa Bouza Vieiro, Leticia Bueno, Juan Bustamante-Munguira, Lucia Cachafeiro, David Campi Hermoso, Sandra Campos Fernández, Iosune Cano, Maria Luisa Cantón-Bulnes, Pablo Cardina Fernández, Laura Carrión García, Sula Carvalho, Núria Casacuberta-Barberà, Manuel Castellà, Andrea Castellví, Pedro Castro, Ramon Cicuendez Ávila, Catia Cillóniz, Luisa Clar, Cristina Climent, Jordi Codina, Pamela Conde, Sofía Contreras, María Cruz Martin, Raul de Pablo Sánchez, Diego De Mendoza, Cecilia del Busto Martínez, Yolanda Díaz, María Digna Rivas Vilas, Cristina Dólera Moreno, Irene Dot, Pedro Enríquez Giraudo, Inés Esmorís Arijón, Teresa Farre Monjo, Javier Fernández, Carlos Ferrando, Albert Figueras, Eva Forcadell-Ferreres, Lorena Forcelledo Espina, Àngels Furro, Felipe García, Beatriz García, Emilio García Prieto, Carlos García Redruello, Amaia García Sagastume, Maria Luisa Gascón Castillo, Gemma Gomà, Vanesa Gómez Casal, Silvia Gómez, Carmen Gómez Gonzalez, Jessica González, Federico Gordo, Maria Pilar Gracia, Alba Herraiz, Rubén Herrán-Monge, Mercedes Ibarz, Silvia Iglesias, Maria Teresa Janer, Gabriel Jiménez, Mar Juan Díaz, Karsa Kiarostami, Juan I. Lazo Álvarez, Miguel León, Alexandre López-Gavín, Ana López Lago, Desire Macias Guerrero, Nuria Mamolar Herrera, Rafael Mañez Mendiluce, Cecilia L Mantellini, Gregorio Marco Naya, Pilar Marcos, Enrique Marmol Peis, Paula Martín Vicente, María Martínez, Carmen Eulalia Martínez Fernández, Maria Dolores Martínez Juan, Juan Fernando Masa Jimenez, Joan Ramon Masclans, Emilio Maseda, Eva María Menor Fernández, Mar Miralbés, Josman Monclou, Juan Carlos Montejo-González, Neus Montserrat, María Mora Aznar, Pedro Moral-Parras, Dulce Morales, Sara Guadalupe Moreno Cano, David Mosquera Rodríguez, Rosana Muñoz-Bermúdez, José María Nicolás, Ramon Nogue Bou, Rafaela Nogueras Salinas, Marta Ocón, Ana Ortega, Sergio Ossa, Pablo Pagliarani, Anna Parera Pous, Francisco Parrilla, Leire Pérez Bastida, Purificación Pérez, Gloria Pérez Planelles, Eva Pérez Rubio, David Pestaña Laguna, Àngels Piñol-Tena, Javier Prados, Andrés Pujol, Núria Ramon Coll, Gloria Renedo Sanchez-Giron, Laura Rodriguez, Felipe Rodríguez de Castro, Silvia Rodríguez, Covadonga Rodríguez Ruiz, Jorge Rubio, Alberto Rubio López, Miriam Ruiz Miralles, Pablo Ryan Murúa, Eva Saborido Paz, Ana Salazar Degracia, Miguel Sanchez, Ana Sánchez, Bitor Santacoloma, Maria Teresa Sariñena, Marta Segura Pensado, Lidia Serra, Mireia Serra-Fortuny, Ainhoa Serrano Lázaro, Lluís Servià, Laura Soliva, Carla Speziale, Daniel Tognetti, Adrián Tormos, Mateu Torres, Sandra Trefler, Javier Trujillano, Luis Urrelo-Cerrón, Estela Val, Luis Valdivia Ruiz, Montse Vallverdú, Maria Van der Hofstadt Martin-Montalvo, Sabela Vara Adrio, Nil Vázquez, Javier Vengoechea, Pablo Vidal Cortes, Clara Vilà-Vilardel, Judit Vilanova, Tatiana Villada Warrington, Hua Yang, Minlan Yang, Ana Zapatero, Júlia Vidal

**Affiliations:** 1 CIBER de Enfermedades Respiratorias (CIBERES), Instituto de Salud Carlos III, Madrid, Spain; 2 Surgical Intensive Care Unit, Anesthesiology, Hospital Clinic of Barcelona, Barcelona, Spain; 3 August Pi i Sunyer Biomedical Research Institute-IDIBAPS, University of Barcelona, Barcelona, Spain; 4 Department of Anesthesiology and Intensive Care, San Bortolo Hospital, Vicenza, Italy; 5 Department of Pathophysiology and Transplantation, Università Degli Studi di Milano, Milan, Italy; 6 Critical Care Center, Institut d’Investigació i Innovació Parc Taulí I3PT, Parc Taulí Hospital Universitari, Sabadell, Spain; 7 Intensive Care Unit, Grupo Quironsalud, Hospital Universitari Sagrat Cor, Barcelona, Spain; 8 Intensive Care Department, Hospital Universitari Vall d’Hebron, Vall d’Hebron Institut de Recerca, Barcelona, Spain; 9 Hospital Universitario de Getafe, Universidad Europea, Madrid, Spain; 10 Dept. of Bioengineering, Universidad Carlos III, Madrid, Spain; 11 Translational Research in Respiratory Medicine, Respiratory Department, Hospital Universitari Aranu de Vilanova and Santa Maria, IRBLleida, Lleida, Spain; 12 Intensive Care Clinical Unit, Hospital Universitario Virgen de Rocío, Seville, Spain; 13 Servicio de Medicina Intensiva, Hospital Universitario La Paz, IdiPAZ, Madrid, Spain; 14 Hospital Universitario San Agustín, Asturias, Spain; 15 Hospital Santa Maria, IRBLleida, Lleida, Spain; 16 Hospital Universitario HM Montepríncipe, Facultad HM Hospitales de Ciencias de la Salud, Universidad Camilo Jose Cela, Madrid, Spain; 17 Servicio de Medicina Intensiva, Hospital Universitario Ramón y Cajal, Madrid, Spain; 18 Intensive Care Unit, and Emergency Medicine, Universidad de Alcalá, Madrid, Spain; 19 Hospital Universitario de Cruces, Barakaldo, Spain; 20 Department of Intensive Care Medicine, Hospital Clínico Universitario Valladolid, Valladolid, Spain; 21 Critical Intensive Medicine Department, Hospital Universitari Arnau de Vilanova de Lleida, IRBLleida, Lleida, Spain; 22 Intensive Care Clinical Unit, Hospital Universitario Virgen Macarena, Seville, Spain; 23 Intensive Care Unit, Hospital Álvaro Cunqueiro, Vigo, Spain; 24 Intensive Care Unit, Hospital Clínico Universitario, Valencia, Spain; 25 Department of Intensive Care Medicine, Hospital Universitario 12 de Octubre, Madrid, Spain; 26 Hospital Universitario de Móstoles, Madrid, Spain; 27 Department of Critical Care Medicine, CHUS, Complejo Hospitalario Universitario de Santiago, Santiago, Spain; 28 Department of Intensive Care, Hospital Universitari de Bellvitge, Bellvitge Biomedical Research Institute (IDIBELL), L’Hospitalet de Llobregat, Barcelona, Spain; 29 Hospital de Mataró de Barcelona, Barcelona, Spain; 30 Department of Medicine, Critical Care Department, Corporació Sanitària Parc Taulí, Universitat Autònoma de Barcelona (UAB), Sabadell, Barcelona, Spain; 31 Department of Medicine, Intensive Care Unit University Hospital of Jerez, University of Cádiz, INIBiCA, Cádiz, Spain; 32 Unidad de Cuidados Intensivos, Hospital Universitario San Pedro de Alcántara, Cáceres, Spain; 33 Hospital General Universitario Gregorio Marañón, Madrid, Spain; 34 Pulmonary and Critical Care Division, Emergency Department, Clínica Sagrada Família, Barcelona, Spain; 35 Intensive Care Department, Hospital Nuestra Señora de Gracia, Saragossa, Spain; 36 Hospital Universitario Virgen de Valme, Unidad de Medicina Intensiva, Seville, Spain; 37 Critical Care Department, Hospital del Mar-IMIM, Barcelona, Spain; 38 Hospital Universitario Torrejón-Universidad Francisco de Vitoria, Madrid, Spain; 39 Department of Intensive Medicine, Hospital Universitario Infanta Leonor, Madrid, Spain; 40 Critical Care Department, Hospital Universitario Lucus Augusti, Lugo, Spain; 41 Complejo Asistencial Universitario de Palencia, Palencia, Spain; 42 Departamento de Biología Funcional, Instituto Universitario de Oncología del Principado de Asturias, Instituto de Investigación Sanitaria del Principado de Asturias, Hospital Central de Asturias, Universidad de Oviedo, Oviedo, Spain; 43 Hospital General de Segovia, Segovia, Spain; 44 Servei de Medicina Intensiva, Hospital Universitari Son Espases, Illes Balears, Palma, Spain; 45 Servicio de Medicina Intensiva, Hospital Universitario Marqués de Valdecilla, Santander, Spain; 46 Servicio de Microbiología Clínica, Facultad de Medicina, Departamento de Biomedicina y Biotecnología, Hospital Universitario Príncipe de Asturias - Universidad de Alcalá, Madrid, Spain; 47 Centro de Investigación Biomédica en Red en Enfermedades Infecciosas (CIBERINFEC), Instituto de Salud Carlos III, Madrid, Spain; 48 Servei de Medicina Intensiva, Hospital Universitari Germans Trias, Badalona, Spain; 49 Critical Care Department, Hospital Universitario Joan XXIII, CIBERES, Rovira & Virgili University, IISPV, Tarragona, Spain; 50 Hospital Universitario de Salamanca, Salamanca, Spain; 51 Intensive Care Unit, Hospital Universitario Sant Joan d’Alacant, Sant Joan d’Alacant, Alicante, Spain; 52 Servicio de Medicina Intensiva, Hospital Universitario y Politécnico La Fe, Valencia, Spain; 53 Institut d’Investigació Sanitària Pere Virgili (IISPV), Hospital Verge de la Cinta, Tortosa, Tarragona, Spain; 54 Intensive Care Unit, Hospital Son Llàtzer, Illes Balears, Palma, Spain; 55 Critical Care Department, Hospital Universitario de GC Dr. Negrín, Universidad Fernando Pessoa Canarias, Las Palmas, Gran Canaria, Spain; 56 Critical Care Department, Hospital Universitario Río Hortega de Valladolid, Valladolid, Spain; 57 Servicio de Medicina Intensiva, Hospital Universitario Mútua de Terrassa, Terrassa, Barcelona, Spain; 58 Servicio de Medicina Intensiva, Hospital Punta de Europa, Algeciras, Spain; 59 Hospital Universitario de León, León, Spain; 60 Complexo Hospitalario Universitario de Ourense, Orense, Spain; 61 Department of Pulmonary Medicine, Hospital Clinic of Barcelona, C/ Villarroel 170, 08036 Barcelona, Spain

**Keywords:** Mechanical power, COVID-19, Mechanical ventilation, Respiratory failure

## Abstract

**Background:**

The relative contribution of the different components of mechanical power to mortality is a subject of debate and has not been studied in COVID-19. The aim of this study is to evaluate both the total and the relative impact of each of the components of mechanical power on mortality in a well-characterized cohort of patients with COVID-19-induced acute respiratory failure undergoing invasive mechanical ventilation. This is a secondary analysis of the CIBERESUCICOVID project, a multicenter observational cohort study including fifty Spanish intensive care units that included COVID-19 mechanically ventilated patients between February 2020 and December 2021. We examined the association between mechanical power and its components (elastic static, elastic dynamic, total elastic and resistive power) with 90-day mortality after adjusting for confounders in seven hundred ninety-nine patients with COVID-19-induced respiratory failure undergoing invasive mechanical ventilation.

**Results:**

At the initiation of mechanical ventilation, the PaO_2_/FiO_2_ ratio was 106 (78; 150), ventilatory ratio was 1.69 (1.40; 2.05), and respiratory system compliance was 35.7 (29.2; 44.5) ml/cmH_2_O. Mechanical power at the initiation of mechanical ventilation was 24.3 (18.9; 29.6) J/min, showing no significant changes after three days. In multivariable regression analyses, mechanical power and its components were not associated with 90-day mortality at the start of mechanical ventilation. After three days, total elastic and elastic static power were associated with higher 90-day mortality, but this relationship was also found for positive end-expiratory pressure.

**Conclusions:**

Neither mechanical power nor its components were independently associated with mortality in COVID-19-induced acute respiratory failure at the start of MV. Nevertheless, after three days, static elastic power and total elastic power were associated with lower odds of survival. Positive end-expiratory pressure and plateau pressure, however, captured this risk in a similar manner.

**Supplementary Information:**

The online version contains supplementary material available at 10.1186/s13613-025-01430-6.

## Background

Ventilator-induced lung injury (VILI) occurs in patients with acute respiratory distress syndrome (ARDS) and increases mortality risk [1, 2]. Excessive strain and stress caused by mechanical ventilation (MV) are the primary mechanisms of lung injury [3]. However, given that VILI is a complex and multifactorial phenomenon [3], a definitive variable to evaluate the risk of lung injury and optimize the application of MV is currently unknown.

Mechanical power is a composite variable that expresses the work that MV transfers to the respiratory system per unit of time. It accounts for the energy transferred to the lungs to produce motion [4]: the first component is the work performed by the ventilator to overcome the basal tension of the lungs produced by positive end-expiratory pressure (PEEP, elastic static power); the second component is the work needed to inflate the lungs, which depends on the elastance of the respiratory system (Ers, elastic dynamic power); the third and final component is the work performed to overcome resistance (R, resistive power). Consequently, mechanical power is a unifying concept of VILI as it considers all variables known to play a role in lung injury, including those previously neglected such as the respiratory rate [4]. For example, simultaneous changes in different ventilator settings will alter these components. However, the final contribution of such changes to VILI risk will be encompassed by mechanical power.

Several observational and experimental studies have identified that mechanical power is associated with increased mortality in critically ill patients, specifically in those with ARDS [5, 6]. However, there are still controversies regarding its role as the ultimate predictor of VILI. Firstly, the relative contribution of each of its components to mortality is debated [6]. Secondly, it has not been studied in patients with COVID-19-induced respiratory failure. Therefore, the aim of this study is to evaluate both the total and the relative impact of each of the components of mechanical power on mortality in a well-characterized cohort of patients with COVID-19-induced acute respiratory failure undergoing invasive MV.

## Methods

### Study design

This is an ancillary analysis of a multi-center, observational cohort study that included patients undergoing invasive MV due to COVID-19. The study involved 50 Spanish intensive care units (ICU) participating in the CIBERESUCICOVID project (NCT04457505) (details of participating centers are provided in Online Table 1). The study was approved by the Institution’s Internal Review Board (Comité Ètic d’Investigació Clínica, registry number HCB/2020/0370, April 2020), and it conducted in accordance with the Helsinki Declaration of 1975, as most recently amended (https://www.wma.net/policies-post/wma-declaration-of-helsinki-ethical-principles-for-medical-research-involving-human-subjects/). Informed consent was obtained from either patients or their relatives. The study spanned from February 6th 2020 to August 16th 2022. During this period, we examined the association between mechanical power at the initiation of MV and after three days with mortality and other clinical outcomes.

**Table 1:** Baseline characteristics of the study population

Characteristic	Population (n = 799)
Age, years	63 (55; 70)
Male sex	567 (71)
BMI, kg/m^2^	29.4 (26.6; 33.3)
Comorbidities^a^	597 (76)
Active smoker	51 (6.7)
Hypertension	419 (52.4)
Diabetes mellitus	206 (25.8)
Dyslipidemia	273 (34.2)
Chronic liver disease	30 (3.8)
Chronic heart disease	109 (13.6)
Chronic lung disease	118 (14.8)
Chronic renal failure	56 (7)
Immunosuppression	44 (5.5)
Glasgow Coma Scale	15 (15; 15)
APACHE-II score	11 (9; 15)
SOFA score	6 (4; 8)
SOFA, hemodynamic component	1 (0; 4)
SOFA, renal component	0 (0; 0)
Arterial blood gases	
PaO_2_/FiO_2_ ratio	106 (78; 150)
pH	7.41 (7.34; 7.45)
PaCO2, mmHg	39 (33.8; 45)

Ventilatory setting and respiratory system mechanics	Upon MV Start	On MV day 3^b^	p-value^b^
Tidal volume/PBW, mL/kg	6.9 (6.2; 7.6)	6.8 (6; 7.7)	0.643
Respiratory rate, breaths per min	22 (20; 25)	22 (20; 26)	0.635
PEEP, cmH_2_O	12 (10; 14)	12 (10; 14)	**0.002**
FiO_2_, %	75 (60; 100)	50 (40; 60)	** < 0.001**
Peak inspiratory pressure, cmH_2_O	32 (29; 36)	31 (29; 35)	0.262
End-inspiratory plateau pressure, cmH_2_O	25 (22; 27)	24 (21; 26)	** < 0.001**
Driving pressure, cmH_2_O	12 (10; 15)	12 (10; 14)	**0.027**
Respiratory system compliance, mL/cmH_2_O	35.7 (29.2; 44.5)	37.4 (30; 45.2)	**0.010**
Ventilatory ratio	1.69 (1.4; 2.05)	1.79 (1.51; 2.17)	** < 0.001**
Total MP, J/min	24.3 (18.9; 29.6)	23.4 (18.5; 29.7)	0.665
Resistive MP, J/min	6.6 (3.8; 10)	6.6 (3.5; 11)	**0.015**
Elastic MP, J/min	16.9 (14; 20.3)	16.1 (13.4; 19.9)	**0.031**
Elastic, static MP, J/min	11.2 (9.1; 13.6)	11 (8.9; 13.5)	**0.027**
Elastic, dynamic MP, J/min	5.5 (4.3; 7.1)	5.2 (4.1; 6.6)	0.107

Data are presented as medians (IQR) or as numbers (%). Percentages calculated on non-missing data. p-values marked in bold indicate statistical significancy on the 95% confidence limit

APACHE: acute physiology and chronic health evaluation; BMI: body mass index; FiO_2_: fraction of inspired oxygen; ICU: intensive care unit; IQR: interquartile range; MP, Mechanical Power; MV: mechanical ventilation; PaCO_2_: arterial partial pressure of carbon dioxide; PaO_2_: arterial partial pressure of oxygen; PBW: predicted body weight; PEEP: positive end-expiratory pressure; SOFA: sequential organ failure assessment

^a^More than one comorbidity possible

^b^Calculated only for patients with MP on day 3 of MV (n = 306)

### Study population

**Fig. 1:**
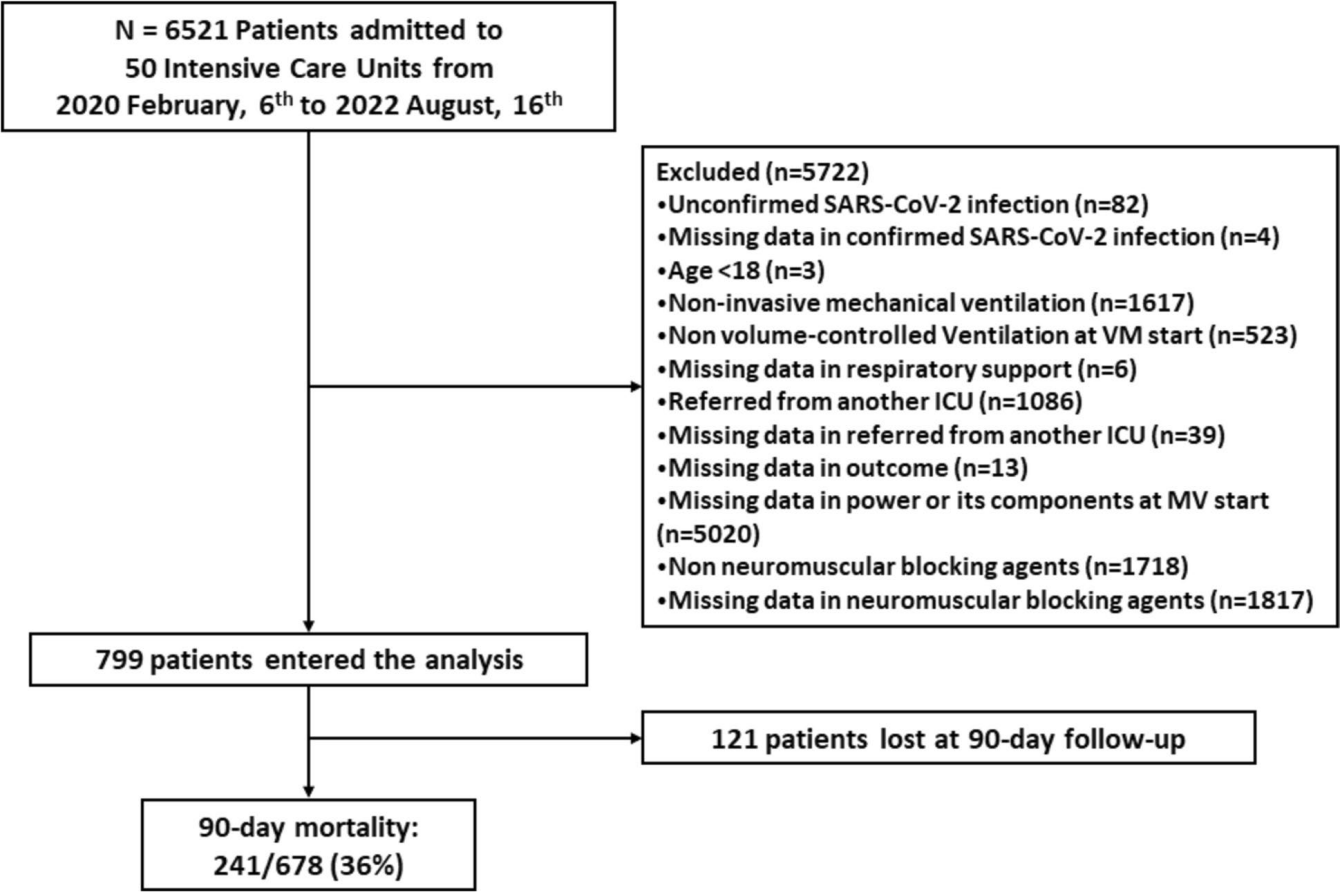
Study flowchart

All patients from the CIBERESUCICOVID project were screened for eligibility. We excluded patients under non-invasive support therapies, receiving pressure-controlled ventilation or any modality other than volume-controlled, patients spontaneously breathing, individuals with missing data for calculating mechanical power or for evaluating clinical outcomes, patients referred from another ICU, < 18 years old, and those lacking a microbiologically confirmed SARS-CoV-2 infection. A total of 121 patients were lost to follow up after 90 days (Fig. 1).

### Exposure variables

The following ventilatory variables were recorded at the initiation of MV and after 3 days: tidal volume [TV, ml and ml/predicted body weight (PBW)], positive end-expiratory pressure [PEEP, cmH_2_O], positive end-inspiratory plateau pressure [Pplat, cmH_2_O], driving pressure [DP, cmH_2_O (Pplat minus PEEP)], peak inspiratory pressure [Ppeak, cmH_2_O], and respiratory rate [RR, breaths/minute]. Respiratory system compliance [ml/cmH_2_O] and ventilatory ratio were calculated as described elsewhere [7, 8]. Mechanical power [J/min] and its components were determined using to the following formula:

$$Resistive \, power\left[ {\text{related to resistance}} \right]: \, 0.0{98 }*{\text{ VT }}*{\text{ RR }}* \, \left( {{\text{Ppeak}}{-}{\text{Pplat}}} \right).$$

$$Elastic \, dynamic \, power\left[ {\text{related to DP}} \right]: \, 0.0{98 }*{\text{ VT }}*{\text{ RR }}* \, \raise.5ex\hbox{$\scriptstyle 1$}\kern-.1em/ \kern-.15em\lower.25ex\hbox{$\scriptstyle 2$} {\text{DP}}.$$

$$Elastic \, static \, power\left[ {\text{related to PEEP}} \right]: \, 0.0{98 }*{\text{ VT }}*{\text{ RR }}*{\text{ PEEP}}.$$

$$Total \, elastic \, power\left[ {\text{related to Pplat}} \right]:{\text{ Elastic dynamic power}}\, + \,{\text{Elastic static power}}.$$

$$Total \, mechanical \, power:{\text{ Resistive power}}\, + \,{\text{Total elastic power}}.$$

### Primary and secondary outcomes

Our primary outcome was 90-day mortality. Secondary outcomes included 30-day mortality, duration of mechanical ventilation, and length of ICU stay. The duration of invasive MV was measured from its initiation until either extubation or death.

### Statistical analyses

Continuous variables are expressed as medians with interquartile ranges (IQR). Categorical variables are expressed as total number and percentage (%). Categorical variables were compared using the chi-squared test or Fisher’s exact test. Continuous variables were compared using the non-parametric Mann–Whitney U test. For comparisons among more than 2 groups, we employed the non-parametric Kruskal–Wallis test. Pairwise comparisons were performed using the Bonferroni method. Continuous paired data (i.e., at the initiation of MV and after 3 days) was compared using the Wilcoxon signed-rank test. We examined the association between mechanical power and its components with 90-day mortality using Cox regression multivariable models [9], adjusting for potential confounders, including COVID-19 wave and center. We adjusted for the following covariates: age, sex, days from symptoms to ICU admission, APACHE-II, PaO_2_/FiO_2_ ratio, pH, static compliance of the respiratory system, ventilatory ratio, prone position and corticosteroids treatment. Results were expressed as hazard ratios (HRs) and 95% confidence interval. Single collinearity was evaluated using Pearson’s correlation coefficient (r). Multicollinearity was assessed using the variance inflation factor (VIF). In the multivariable Cox regression models we examined whether mechanical power, each of its components (elastic static power, elastic dynamic power and resistive power), and total elastic power were associated with mortality after the inclusion of covariates. We repeated these analyses with data from the third day after MV initiation. Finally, we performed the same analyses using ventilatory parameters other than power and compared their ability, along with power, to predict mortality using the receiver operating characteristic (ROC) curve. The multiple imputation method was employed for handling missing data in the covariates of the multivariable analyses [10, 11]. The significance level was set at 0.05 (two-tailed), and all analyses were conducted using IBM SPSS version 26.0 (IBM Corp., Armonk, NY, USA).

## Results

### Characteristics of the study population

Out of 6.521 patients were screened for eligibility, 5.722 patients did not meet inclusion criteria. The final analysis included a total of 799 patients with COVID-19-induced acute respiratory failure undergoing invasive MV [Fig. 1]. 27 and 19 out of 799 patients died or were transferred before day 3.

The baseline characteristics of the study population are summarized in Table 1. The median age was 63 years (55; 70), and 567 (71%) patients were male. Upon ICU admission, the median APACHE-II and SOFA scores were 11 (9; 15) and 6 (4; 8), respectively. The median PaO_2_/FiO_2_ was 106 (78; 150) at the initiation of MV. Ventilatory ratio was1.69 (1.40; 2.05) at the initiation of MV and 1.79 (1.51; 2.17) after 3 days of MV (p < 0.001). Respiratory system compliance increased from 35.7 (29.2; 44.5) ml/cmH_2_O at the initiation of MV to 37.4 (30; 45.2) ml/cmH_2_O after 3 days of MV (p = 0.014).

In general, a lung-protective ventilation strategy was implemented for patients in this cohort. The median tidal volume and PEEP were 6.9 (6.2; 7.6) mL/kg PBW and 12 (10; 14) cmH_2_O at the initiation of MV, similar to that applied after three days. Mechanical power was 23.8 (18.8; 28.9) J/min at MV onset, with no significant changes observed after three days. Resistive, elastic static, elastic dynamic as well as total elastic and total power at MV start and after three days are shown in Table 1.

### Outcomes according to mechanical power and other ventilator-induced lung injury parameters

Table 2 presents the characteristics of survivors vs. deceased patients. Overall, 241 (35.5%) patients died within the 90-day follow-up. Non-survivors were older [67 (60; 74) vs 62 (53; 69) years, p < 0.001], and had higher APACHE-II [10 (8; 13) vs 13 (10; 18), p < 0.001] and SOFA scores [6 (4; 8) vs 7 (4; 8), p < 0.001,] upon ICU admission. Non-survivors had a higher ventilatory ratio [1.62 (1.34; 1.99) vs 1.74 (1.46; 2.26), p < 0.001]. Regarding ventilatory settings and pulmonary mechanics, non-survivors presented with a slightly higher driving pressure [12 (10; 14) vs. 13 (10; 15) cmH_2_O, p = 0.009], and mildly lower respiratory system compliance [36.1 (30; 45) vs. 33.6 (27.1; 42.3) mL/cmH_2_O, p = 0.006]. Upon ICU admission, survivors had a similar median mechanical power [23.8 (18.6; 29.1) vs. 24.6 (19.4; 31) J/min, p = 0.152] compared to non-survivors. However, elastic dynamic power was lower in survivors [5.4 (4.3; 6.9) vs 5.9 (4.4; 7.5) J/min, p = 0.013]. On day three, mechanical power and most of its components were higher in non-survivors (Table 2).

**Table 2:** Characteristics of patients and outcomes based on 90-day mortality in the overall population

Variables	Survivors (n = 437)	Non-survivors (n = 241)	p-value
Age, years	62 (53; 69)	67 (60; 74)	** < 0.001**
Male sex	309 (70.7)	177 (73.4)	0.449
APACHE-II score	10 (8; 13)	13 (10; 18)	** < 0.001**
SOFA score	6 (4; 8)	7 (4; 8)	** < 0.001**
PaO_2_/FiO_2_ ratio	107 (80; 159)	102 (74.6; 140)	0.084
pH	7.42 (7.35; 7.46)	7.38 (7.29; 7.45)	** < 0.001**
PaCO_2_, mmHg	39 (33.8; 45)	40.1 (34; 47)	0.154
Ventilatory ratio	1.62 (1.34; 1.99)	1.74 (1.46; 2.26)	**0.003**
Ventilatory setting and pulmonary mechanics on MV start			
Tidal volume/PBW, mL/kg	6.9 (6.2; 7.6)	6.8 (6.1; 7.6)	0.687
Respiratory rate, breaths per min	22 (20; 25)	22 (20; 25)	0.215
PEEP, cmH_2_O	12 (11; 14)	12 (10; 14)	0.362
Peak inspiratory pressure, cmH_2_O	31 (29; 35)	33 (29; 37)	**0.004**
End-inspiratory plateau pressure, cmH_2_O	25 (22; 27)	25 (23; 28)	**0.021**
Driving pressure, cmH_2_O^a^	12 (10; 14)	13 (10; 15)	**0.009**
Compliance, mL/cmH_2_O	36.1 (30; 45)	33.6 (27.1; 42.3)	**0.006**
Total MP, J/min	23.8 (18.6; 29.1)	24.6 (19.4; 31)	0.152
Resistive MP, J/min	6.2 (3.5; 9.5)	6.5 (3.7; 10.3)	0.250
Elastic MP, J/min	17 (14; 20.2)	17.4 (14.1; 21.4)	0.250
Elastic, Static MP, J/min	11.2 (9.3; 13.8)	11.5 (9; 14)	0.661
Elastic, Dynamic MP, J/min	5.4 (4.3; 6.9)	5.9 (4.4; 7.5)	**0.013**
Ventilatory setting and pulmonary mechanics on MV day 3^b^			
Tidal volume/PBW, mL/kg	6.9 (6.1; 7.7)	6.7 (6; 7.4)	0.602
Respiratory rate, breaths per min	22 (20; 25)	24(20; 27)	**0.008**
PEEP, cmH_2_O	12 (10; 14)	12 (10; 14)	0.601
Peak inspiratory pressure, cmH_2_O	30 (29; 34)	32.2 (29; 36)	**0.029**
End-inspiratory plateau pressure, cmH_2_O	24 (21; 26)	25 (23; 28)	**0.005**
Driving pressure, cmH_2_O	11 (10; 13)	12 (10; 15)	**0.015**
Compliance, mL/cmH_2_O	38.2 (30.8; 46.3)	35 (28.7; 42.5)	**0.037**
Total MP, J/min	21.8 (18; 29.4)	26.5 (19.4; 32.4)	**0.034**
Resistive MP, J/min	5.9 (3.3; 11.2)	7.2 (3.2; 11.4)	0.657
Elastic MP, J/min	15.7 (13.3; 19.5)	18 (14.4; 22.2)	**0.004**
Elastic, static MP, J/min	10.9 (9.1; 13.4)	11.8 (9.5; 14.9)	**0.059**
Elastic, dynamic MP, J/min	5 (3.9; 6.5)	5.9 (4.7; 7.4)	**0.001**
Outcomes			
30-day mortality^c^	177 (24.1)		
90-day mortality^a^	241 (35.5)		
Length of ICU stay, days^e^	22 (13; 39)		
Length of hospital stay, days^e^	37 (23; 54)		
Invasive MV length, days^e^	16 (9; 31.5)		

Data are presented as medians (IQR) or as numbers (%). Percentages calculated on non-missing data. p-values marked in bold indicate statistical significancy on the 95% confidence limit

APACHE: acute physiology and chronic health evaluation; IQR: interquartile range; MP, Mechanical Power; MV: mechanical ventilation; PaCO_2_: arterial partial pressure of carbon dioxide; PaO_2_: arterial partial pressure of oxygen; PBW: predicted body weight; PEEP: positive end-expiratory pressure; SOFA: sequential organ failure assessment

^a^Calculated only for patients with 90-day follow-up (n = 678)

^b^Calculated only for patients with MP data on MV day 3 (n = 306)

^c^Calculated only for patients with 30-day follow-up (n = 735)

^e^Calculated only for surviving patients (n = 437)

Potential predictors of 90-day mortality were evaluated in three different multivariable models: Model A included total mechanical power, Model B included total elastic and resistive power, and Model C included elastic static, elastic dynamic and resistive power. After adjusting for age, sex, days from symptoms to ICU admission, APACHE-II, PaO_2_/FiO_2_ ratio, pH, static compliance of the respiratory system, ventilatory ratio, prone position and corticosteroids treatment, none of the three models detected an association between mechanical power or its components and 90-day mortality at the initiation of MV (Table 3). However, total elastic power and static power measured after three days of MV were related to higher mortality (Online Table 3). When assessing the discriminatory ability for mortality using PEEP and Pplat, compared to elastic static power and total elastic power, the areas under the curve were similar for Pplat and total elastic power (p = 0.921), and for PEEP and elastic static power (p = 0.07) (Online Figs. 1 and 2).

**Table 3:** Multivariable models evaluating predictors of 90-day mortality, using total mechanical power and its components

Predictor Variables	HR (95% CI)	p-value
MODEL A		
Age (+ 1 year)^a^	1.05 (1.03 to 1.06)	** < 0.001**
Male sex	1.12 (0.80 to 1.57)	0.511
Days from initial symptoms to ICU admission (+ 1 day)^a^	0.99 (0.97 to 1.02)	0.666
APACHE-II score at ICU admission (+ 1)^a^	1.04 (1.01 to 1.07)	**0.012**
PaO_2_/FiO_2_ ratio at ICU admission (+ 1)^a^	1.00 (1.00 to 1.00)	0.373
pH at ICU admission (+ 1)^a^	0.27 (0.06 to 1.24)	0.091
Compliance at MV start (+ 1 mL/cmH_2_O)^a^	0.99 (0.98 to 1.01)	0.353
Ventilatory ratio at MV start (+ 1)^a^	0.87 (0.67 to 1.11)	0.265
Prone position at MV start	1.03 (0.76 to 1.40)	0.858
MP at MV start (+ 1 J/min)^a^	1.01 (0.99 to 1.04)	0.325
Corticosteroid treatment	0.88 (0.53 to 1.459)	0.609
MODEL B		
Age (+ 1 year)^a^	1.05 (1.03 to 1.06)	** < 0.001**
Male sex	1.11 (0.78 to 1.57)	0.555
Days from initial symptoms to ICU admission (+ 1 day)^a^	0.99 (0.97 to 1.02)	0.612
APACHE-II score at ICU admission (+ 1)^a^	1.05 (1.02 to 1.08)	**0.002**
PaO_2_/FiO_2_ ratio at ICU admission (+ 1)^a^	1.00 (1.00 to 1.00)	0.307
pH at ICU admission (+ 1)^a^	0.38 (0.08 to 1.84)	0.231
Compliance at MV start (+ 1 mL/cmH_2_O)^a^	0.99 (0.98 to 1.01)	0.363
Ventilatory ratio at MV start (+ 1)^a^	0.85 (0.66 to 1.10)	0.225
Prone position at MV start	1.03 (0.76 to 1.41)	0.833
Elastic MP at MV start (+ 1 J/min)^a^	1.01 (0.97 to 1.05)	0.703
Resistive MP at MV start (+ 1 J/min)^a^	1.01 (0.98 to 1.05)	0.442
Corticosteroid treatment	0.90 (0.55 to 1.50)	0.694
MODEL C		
Age (+ 1 year)^a^	1.05 (1.04 to 1.06)	** < 0.001**
Male sex	1.17 (0.82 to 1.67)	0.387
Days from initial symptoms to ICU admission (+ 1 day)^a^	1.00 (0.97 to 1.02)	0.737
APACHE-II score at ICU admission (+ 1)^a^	1.05 (1.02 to 1.08)	**0.001**
PaO_2_/FiO_2_ ratio at ICU admission (+ 1)^a^	1.00 (1.00 to 1.00)	0.278
pH at ICU admission (+ 1)^a^	0.38 (0.08 to 1.84)	0.229
Compliance at MV start (+ 1 mL/cmH_2_O)^a^	0.98 (0.97 to 1.00)	0.06
Ventilatory ratio at MV start (+ 1)^a^	0.88 (0.68 to 1.14)	0.348
Prone position at MV start	1.03 (0.76 to 1.40)	0.841
Elastic, static MP at MV start (+ 1 J/min)^a^	1.04 (0.99 to 1.10)	0.137
Elastic, dynamic MP at MV start (+ 1 J/min)^a^	0.92 (0.83 to 1.02)	0.131
Resistive MP at MV start (+ 1 J/min)^a^	1.02 (0.98 to 1.06)	0.373
Corticosteroid treatment	0.93 (0.56 to 1.53)	0.764

*MODEL A*: using the continuous values of mechanical power at MV start; *MODEL B:* using the continuous values of the elastic and resistive components at MV start simultaneously; *MODEL C*: using the continuous values of the elastic static, elastic dynamic and resistive components at MV start simultaneously. Data are shown as estimated HRs (95% CIs) of the explanatory variables in the 90-day mortality group. The p-value is based on the null hypothesis that all HRs relating to an explanatory variable equal unity (no effect). p-values marked in bold indicate statistical significancy on the 95% confidence limit

APACHE, acute physiology and chronic health evaluation; CI, confidence interval; FiO_2_, fraction of inspired oxygen; HR, hazard ratio; MP, mechanical power; MV, mechanical ventilation; PaO_2_, partial pressure of arterial oxygen

^a^ “ + 1” means a one-unit increase on the scale in the predictor variable

Table 4 presents the association between quintiles of mechanical power at the start of MV with mortality and other secondary outcomes. The fifth quintile of elastic dynamic power was associated with an increase in 90-day compared to the second and third quintile. This association was not significant after multivariable adjustment (Online Table 7).

**Table 4:** Effects of quantiles of Mechanical Power on outcomes, length of ICU stay, and duration of mechanical ventilation

Total MP	Q1 (n = 159)	Q2 (n = 160)	Q3 (n = 160)	Q4 (n = 160)	Q5 (n = 160)	p-value
90-day mortality^a^	44 (32.1)	48 (34)	45 (34.6)	45 (35.2)	59 (41.5)	0.536
30-day mortality^b^	31 (20.8)	33 (22.3)	36 (24.3)	33 (24.1)	44 (28.8)	0.563
Length of ICU stay, days^c^	24 (15; 37)	16 (11; 34)	26 (14; 38)	20 (12; 43)	29 (15; 44)^d^	**0.012**
Invasive MV length, days^c^	18 (11; 29)	12 (8; 24)	19 (9; 32)	16 (7; 35)	21 (9; 34)^d^	**0.019**

Elastic MP	Q1 (n = 159)	Q2 (n = 160)	Q3 (n = 159)	Q4 (n = 163)	Q5 (n = 158)	p-value
90-day mortality^a^	54 (44.66)	41 (29.1)	45 (33.6)	45 (32.8)	56 (38.6)	0.085
30-day mortality^b^	36 (26.3)	33 (21.3)	36 (25.4)	33 (22.6)	39 (25.2)	0.841
Length of ICU stay, days^c^	18 (13; 33)	22 (13; 39)	22 (12; 37)	23 (12; 42)	29 (15; 43)	0.171
Invasive MV length, days^c^	13 (8; 26)	16 (9; 31)	15 (8; 31)	17 (9; 33)	21 (11; 34)	0.137

Elastic, static MP	Q1 (n = 160)	Q2 (n = 160)	Q3 (n = 160)	Q4 (n = 159)	Q5 (n = 160)	p-value
90-day mortality^a^	54 (44.6)	41 (29.1)	45 (33.6)	45 (32.8)	56 (38.6)	0.085
30-day mortality^b^	36 (26.3)	33 (21.3)	36 (25.4)	33 (22.6)	39 (25.2)	0.841
Length of ICU stay, days^c^	18 (13; 33)	22 (13; 39)	22 (12; 37)	23 (12; 42)	29 (15; 43)	0.193
Invasive MV length, days^c^	13 (8; 26)	16 (9; 31)	15 (8; 31)	17 (9; 33)	21 (11; 34)	0.071

Elastic, dynamic MP	Q1 (n = 159)	Q2 (n = 160)	Q3 (n = 160)	Q4 (n = 159)	Q5 (n = 161)	p-value
90-day mortality^a^	47 (36.2)	36 (26.3)	44 (30.8)	52 (39.4)	62 (45.6)^d^	**0.009**
30-day mortality^b^	34 (23.1)	31 (21.1)	30 (20)	39 (27.5)	43 (28.9)	0.297
Length of ICU stay, days^c^	21 (14; 33)	20 (12; 38)	23 (13; 37)	21 (14; 43)	30 (13; 49)	0.460
Invasive MV length, days^c^	15 (9; 27)	16 (8; 28)	16 (9; 29)	15 (9; 32)	20 (9; 40)	0.693

Resistive, MP	Q1 (n = 158)	Q2 (n = 162)	Q3 (n = 160)	Q4 (n = 159)	Q5 (n = 160)	p-value
90-day mortality^a^	50 (34.2)	46 (32.6)	48 (35.6)	44 (34.4)	53 (41.4)	0.622
30-day mortality^b^	34 (21.9)	31 (20.7)	38 (25.9)	34 (24.3)	40 (28)	0.594
Length of ICU stay, days^c^	25 (16; 38)	21 (12; 38)	20 (13; 37)	22 (11; 42)	22 (14; 40)	0.454
Invasive MV length, days^c^	38 (28; 54)	32 (21; 50)	32 (22; 52)	37 (21; 59)	38 (24; 56)	0.382

Data are presented as medians (IQR) or as numbers (%). Percentages calculated on non-missing data. p-values marked in bold indicate statistical significancy on the 95% confidence limit

ICU, intensive care unit; IQR, interquartile range; MP, mechanical power; MV, mechanical ventilation; Q1, MP first quintile; Q2, MP second quintile; Q3, MP third quintile; Q4, MP fourth quintile; Q5, MP fifth quintile

^a^Calculated only for patients with 90-day follow-up (n = 678)

^b^Calculated only for patients with 30-day follow-up (n = 735)

^c^Calculated only for surviving patients (n = 437)

^d^p < 0.05 for comparison with Q2 (Bonferroni correction)

^e^p < 0.05 for comparison with Q3(Bonferroni correction)

## Discussion

In this large multicenter cohort study of patients with COVID-19-induced acute respiratory failure who received lung-protective ventilation, we aimed to evaluate the association of mechanical power and its components with survival. The main findings of this study are as follows: first, at the start of MV, neither mechanical power nor any of its components were independently associated with mortality. Second, after three days of MV, although higher elastic static and total elastic power were associated with lower odds of survival, simpler variables such as PEEP and Pplat captured this increased risk in a similar manner. Overall, these findings suggest that mechanical power and its components have limited additional value in guiding mechanical ventilation in this population.

To the best of our knowledge, this is the largest study analyzing mechanical power in critically ill COVID-19 patients undergoing invasive MV. However, the association between mechanical power components and mortality had previously been investigated in large cohorts of non-COVID-19-induced ARDS [6]. In contrast to our study, they found that the association between mechanical power and mortality was primarily influenced by elastic dynamic power (related to respiratory elastance or driving pressure). In their investigation, respiratory rate was independently associated with mortality, highlighting the additional value of mechanical power in contrast to conventional VILI parameters. When we examined elastic dynamic power both at the initiation of MV and after three days, we found no association with mortality. The same results persisted when we divided the population into quintiles, considering those patients at a higher risk of VILI. Two significant differences might explain the dissociation between both studies. First and foremost, a substantial part of the patients included in their cohort were not subjected to the current standards of lung-protective MV [6]. Consequently, in their study, the median driving pressure was notably higher, and the elastic dynamic power was doubled compared to ours. In contrast, driving pressure was clearly within lung-protective ranges in most patients from our cohort. Other authors who also analyzed respiratory mechanics in patients with COVID-19 found a similar distribution of driving pressure, even in patients with tidal volumes higher than 6 mL/kg PBW [12]. This might reflect clinicians' awareness of the potential harm caused by dynamic strain, leading to a concomitant decrease in elastic dynamic power. Although less probable, the second explanation could be the different etiology of respiratory failure. It is unknown whether conditions other than COVID-19 are more vulnerable to VILI.

One important finding of our study is that both total elastic power and elastic static power, measured on day 3 after the start of MV, were associated with a higher risk of mortality. As we did not find any association with dynamic elastic power, and considering that total elastic power includes both static and dynamic components, these results suggest that static power might contribute to lung damage over time. This finding has not been reported in previous studies analyzing the components of mechanical power, probability because they only analyzed power at MV start [6]. Within the framework of power, higher PEEP could contribute to lung damage as it would increase the basal tension of lung fibers and the work performed by MV during insufflation as a result. However, PEEP limits the harm associated to an expiratory phenomenon (atelectrauma) [13], and power only accounts for events occurring during inspiration. In this experimental study [14], the relationship between PEEP-derived power and lung damage was U-shaped, reinforcing that both atelectrauma and overdistension are harmful. In our study, ventilatory ratio increased after three days of MV, a phenomenon that has been associated with the occurrence of fibroproliferative changes [15]. Consequently, it is possible that most patients tended to develop hyperinflation rather than recruitment with higher PEEP over time [16], limiting its effectiveness in preventing lung damage or even causing harm. Although static elastic power and total elastic power were found to be associated with mortality, simpler variables such as PEEP and Pplat showed similar discriminatory ability, thus making it difficult to incorporate such complex variables into clinical practice for guiding the application of MV.

The association between (total) mechanical power and mortality has also been investigated in other cohorts of mechanically ventilated patients, with or without respiratory failure. Several studies have reported an association between higher mechanical power and worse clinical outcomes in a wide variety of critically ill patients. The association between mechanical power and survival has been reported in patients with acute brain injury [17], pediatric populations [18], surgical patients [19], patients with COVID-19-induced acute respiratory failure [20] and in general ICU cohorts [5]. Similar than the study by Costa et al., most of the studies were conducted in cohorts of patients that were not treated with lung protective MV and, moreover, the association between power and survival was not adjusted for relevant respiratory variables other than PaO_2_/FiO_2_, such as compliance and ventilatory ratio [5, 17–20].

Similar to other studies [6], we did not find resistive power to be associated with mortality. Other authors have shown that inspiratory flow or strain rate can increase lung injury [21]. However, most of the resistive power is absorbed by the endotracheal tube and the upper airways [22], playing a limited role in lung injury.

The strengths of this study are its multicenter nature; the inclusion of patients across different periods (four waves); the addition of relevant respiratory physiological covariates in multivariable regression models; the inclusion of total elastic power; and the performance of multivariable analysis on day 1 of MV and at day 3; finally, the mechanical ventilation practices in the CIBERESUCICOVID study are similar to those of contemporary ARDS cohorts [23], easing the extrapolation of the results to other types of ARDS. Our study has several limitations. First, it is a retrospective study and a significant number of eligible patients had to be excluded due to missing data necessary for calculating mechanical power. This might hamper the generalization of the results. Second, we lack data on intrinsic and total PEEP. Third, despite careful adjustment, we cannot rule out the presence of residual confounders.

## Conclusions

Mechanical power and its components were not independently associated with mortality in COVID-19-induced acute respiratory failure at the start of MV. Nevertheless, after three days, static elastic power and total elastic power were associated with lower odds of survival. PEEP and Pplat, however, captured this risk in a similar manner. These results challenge the use of power and its components for clinical decision-making in this population. Further studies investigating the relationship between power components and mortality are warranted in patients with ARDS other than COVID-19, especially those treated with lung-protective strategies.

## Supplementary Information

Supplementary material 1.

## Data Availability

The datasets used and/or analysed during the current study are available from the corresponding author on reasonable request.

## Abbreviations

APACHE-II: Acute physiology and chronic health disease classification system II
ARDS: Acute respiratory distress syndrome
DP: Driving pressure
Ers: Elastance of the respiratory system
HR: Hazard ratio
ICU: Intensive care unit
IQR: Interquartile range
MP: Mechanical power
MV: Mechanical ventilation
PBW: Predicted body weight
PEEP: Positive end-expiratory pressure
Ppeak: Peak inspiratory pressure
Pplat: Positive end-inspiratory plateau pressure
R: Resistive power
RR: Respiratory rate
SOFA: Sequential organ failure assessment
TV: Tidal volume
VIF: Variance inflation factor
VILI: Ventilator-induced lung injury
