# Supplementary material for: Mechanical power is not associated with mortality in COVID-19 mechanically ventilated patients
Source: Ann Intensive Care. 2025 Feb 25;15:27. doi: 10.1186/s13613-025-01430-6 (PMC11861844; doi:10.1186/s13613-025-01430-6)

## ANNALS OF INTENSIVE CARE

Online Supplement of

**Mechanical power and mortality in COVID-19 mechanically ventilated patients Authors:**

Enric Barbeta 1, 2, 3, Cláudia Barreiros 2, Edoardo Forin 4, Amedeo Guzzardella 5, Anna Motos 1, 3,

Laia Fernández-Barat 1, 3, Albert Gabarrús 1,3, Adrián Ceccato 1, 6, 7, Ricard Ferrer 8, Jordi Riera 8,

Oscar Peñuelas 1, 9, José Ángel Lorente 1, 9, 10, David de Gonzalo-Calvo 1,11, Jessica Gonzalez 1,11, Rosario Amaya-Villar 12, José Manuel Añón 1,13, Ana Balan Mariño 14, Carme Barberà 15, José Barberán 16, Aaron Blandino Ortiz 17, 18, Maria Victoria Boado 19, Elena Bustamante-Munguira 1 20, Jesús Caballero 21, María Luisa Cantón-Bulnes 22, Cristina Carbajales Pérez 23, Nieves Carbonell 24, Mercedes Catalán-González 25, Nieves Franco 26, Cristóbal Galbán 27, Víctor D Gumucio- Sanguino 28, Maria Del Carmen de la Torre 29, Emilio Díaz 30, Ángel Estella 31, Elena Gallego Curto 1 32, José Manuel Gómez 33, Arturo Huerta 34, Ruth Noemí Jorge García 35, Ana Loza-Vázquez 36, Judith Marin-Corral 37, María Cruz Martin Delgado 38, Amalia Martínez de la Gándara 39, Ignacio Martínez Varela 40, Juan Lopez Messa 41, Guillermo M Albaiceta 1,42, María Teresa Nieto 43, Mariana Andrea Novo 44, Yhivian Peñasco 45, Felipe Pérez-García 46, 47, Pilar Ricart 48, Alejandro

Rodríguez 49, Victor Sagredo 50, Angel Sánchez-Miralles 51, Susana Sancho Chinesta 52, Ferran

Roche-Campo 53, Lorenzo Socias 54, Jordi Solé-Violan 1, 55, Luis Tamayo Lomas 1, 56, José Trenado

57, Alejandro Úbeda 58, Luis Jorge Valdivia 59, Pablo Vidal 60, Ferran Barbé 1, 11, Jordi Vallverdú 2, Antoni Torres 1, 60; CIBERESUCICOVID Project investigators (COV20/00110, ISCIII)

**Affiliations:**

1. CIBER de Enfermedades Respiratorias (CIBERES), Instituto de Salud Carlos III, Madrid, Spain.
2. Surgical Intensive Care Unit, Anesthesiology, Hospital Clinic of Barcelona, Barcelona, Spain.

3 August Pi i Sunyer Biomedical Research Institute-IDIBAPS, University of Barcelona, Barcelona, Spain.

1. Department of Anesthesiology and Intensive Care, San Bortolo Hospital, Vicenza, Italy
2. Department of Pathophysiology and Transplantation, Università degli studi di Milano, Milan, Italy.
3. Critical Care Center, Institut d'Investigació i Innovació Parc Taulí I3PT, Parc Taulí Hospital Universitari, Sabadell, Spain.
4. Intensive Care Unit, Grupo Quironsalud, Hospital Universitari Sagrat Cor, Barcelona, Spain.
5. Intensive Care Department, Hospital Universitari Vall d'Hebron, Vall d'Hebron Institut de Recerca, Barcelona, Spain.
6. Hospital Universitario de Getafe, Universidad Europea, Madrid, Spain.
7. Dept. of Bioengineering, Universidad Carlos III, Madrid, Spain.
8. Translational Research in Respiratory Medicine, Respiratory Department, Hospital Universitari Aranu de Vilanova and Santa Maria, IRBLleida, Lleida, Spain.
9. Intensive Care Clinical Unit, Hospital Universitario Virgen de Rocío, Sevilla, Spain.
10. Servicio de Medicina Intensiva, Hospital Universitario La Paz, IdiPAZ, Madrid, Spain.
11. Hospital Universitario San Agustín, Asturias, Spain.
12. Hospital Santa Maria, IRBLleida, Lleida, Spain.
13. Hospital Universitario HM Montepríncipe, Facultad HM Hospitales de Ciencias de la Salud, Universidad Camilo Jose Cela, Madrid, Spain.
14. Servicio de Medicina Intensiva, Hospital Universitario Ramón y Cajal, Madrid, Spain.
15. Intensive Care Unit, and Emergency Medicine, Universidad de Alcalá, Madrid, Spain.
16. Hospital Universitario de Cruces, Barakaldo, Spain.
17. Department of Intensive Care Medicine, Hospital Clínico Universitario Valladolid, Valladolid, Spain.
18. Critical Intensive Medicine Department, Hospital Universitari Arnau de Vilanova de Lleida, IRBLleida,

Lleida, Spain.

1. Intensive Care Clinical Unit, Hospital Universitario Virgen Macarena, Seville, Spain.
2. Intensive Care Unit, Hospital Álvaro Cunqueiro, Vigo, Spain.
3. Intensive Care Unit, Hospital Clínico Universitario, Valencia, Spain.
4. Department of Intensive Care Medicine, Hospital Universitario 12 de Octubre, Madrid, Spain.
5. Hospital Universitario de Móstoles, Madrid, Spain.
6. Department of Critical Care Medicine, CHUS, Complejo Hospitalario Universitario de Santiago, Santiago, Spain.
7. Department of Intensive Care, Hospital Universitari de Bellvitge, Bellvitge Biomedical Research Institute (IDIBELL), L'Hospitalet de Llobregat, Barcelona, Spain.
8. Hospital de Mataró de Barcelona, Barcelona, Spain.
9. Department of Medicine, Critical Care Department, Corporació Sanitària Parc Taulí, Universitat Autònoma de Barcelona (UAB), Sabadell, Barcelona, Spain.
10. Department of Medicine, Intensive Care Unit University Hospital of Jerez, University of Cádiz, INIBiCA, Cádiz, Spain.
11. Unidad de Cuidados Intensivos, Hospital Universitario San Pedro de Alcántara, Cáceres, Spain.
12. Hospital General Universitario Gregorio Marañón, Madrid, Spain.
13. Pulmonary and Critical Care Division, Emergency Department, Clínica Sagrada Família, Barcelona, Spain.
14. Intensive Care Department, Hospital Nuestra Señora de Gracia, Zaragoza, Spain.
15. Unidad de Medicina Intensiva, Hospital Universitario Virgen de Valme, Seville, Spain.
16. Critical Care Department, Hospital del Mar-IMIM, Barcelona, Spain.
17. Hospital Universitario Torrejón-Universidad Francisco de Vitoria, Madrid, Spain.
18. Department of Intensive Medicine, Hospital Universitario Infanta Leonor, Madrid, Spain.
19. Critical Care Department, Hospital Universitario Lucus Augusti, Lugo, Spain.
20. Complejo Asistencial Universitario de Palencia, Palencia, Spain.
21. Departamento de Biología Funcional, Instituto Universitario de Oncología del Principado de Asturias, Instituto de Investigación Sanitaria del Principado de Asturias, Hospital Central de Asturias, Universidad de Oviedo, Oviedo, Spain.
22. Hospital General de Segovia, Segovia, Spain.
23. Servei de Medicina Intensiva, Hospital Universitari Son Espases, Illes Balears, Palma, Spain.
24. Servicio de Medicina Intensiva, Hospital Universitario Marqués de Valdecilla, Santander, Spain.
25. Servicio de Microbiología Clínica, Hospital Universitario Príncipe de Asturias - Universidad de Alcalá,

Facultad de Medicina, Departamento de Biomedicina y Biotecnología, Madrid, Spain.

1. Centro de Investigación Biomédica en Red en Enfermedades Infecciosas (CIBERINFEC), Instituto de Salud Carlos III, Madrid, Spain.
2. Servei de Medicina Intensiva, Hospital Universitari Germans Trias, Badalona, Spain.
3. Critical Care Department, Hospital Universitario Joan XXIII, CIBERES, Rovira & Virgili University, IISPV, Tarragona, Spain.
4. Hospital Universitario de Salamanca, Salamanca, Spain.
5. Intensive Care Unit, Hospital Universitario Sant Joan d'Alacant, Sant Joan d'Alacant, Alicante, Spain.
6. Servicio de Medicina Intensiva, Hospital Universitario y Politécnico La Fe, Valencia, Spain.
7. Hospital Verge de la Cinta, Institut d'Investigació Sanitària Pere Virgili (IISPV), Tortosa, Tarragona, Spain.
8. Intensive Care Unit, Hospital Son Llàtzer, Illes Balears, Palma, Spain.
9. Critical Care Department, Hospital Universitario de GC Dr. Negrín, Universidad Fernando Pessoa Canarias,

Las Palmas, Gran Canaria, Spain.

1. Critical Care Department, Hospital Universitario Río Hortega de Valladolid, Valladolid, Spain.
2. Servicio de Medicina Intensiva, Hospital Universitario Mútua de Terrassa, Terrassa, Barcelona, Spain.
3. Servicio de Medicina Intensiva, Hospital Punta de Europa, Algeciras, Spain.
4. Hospital Universitario de León, León, Spain.
5. Complexo Hospitalario Universitario de Ourense, Orense, Spain.
6. Department of Pneumology, Hospital Clinic of Barcelona, Barcelona, Spain.

**INDEX**

[Online Table 1. List of participating centers 7](#_bookmark0)

[Online Table 2. Quintiles of MP and its components at MV start 9](#_bookmark1)

[Online Table 3. Effects of quintiles of MP and its components on outcomes, length of ICU stay, and duration of mechanical](#_bookmark2) [ventilation (day 3 of mechanical ventilation) 10](#_bookmark2)

[Online Table 4. Multivariable model assessing predictors of 90-day mortality, using the](#_bookmark3) [continuous values of MP at day 3 of MV 12](#_bookmark3)

[Online Table 5. Multivariable model assessing predictors of 90-day mortality, using the](#_bookmark4) [quintiles values of MP at MV start 14](#_bookmark4)

[Online Table 6. Multivariable model assessing predictors of 90-day mortality, using the](#_bookmark5) [quintiles values of elastic and resistive MP components at MV start simultaneously 15](#_bookmark5)

[Online Table 7. Multivariable model assessing predictors of 90-day mortality, using the](#_bookmark6) [quintiles values of elastic static, elastic dynamic and resistive MP components at MV start](#_bookmark6) [simultaneously 16](#_bookmark6)

[Online Table 8. Multivariable model assessing predictors of 90-day mortality, using the](#_bookmark7) [quintiles values of the MP at day 3 of MV 18](#_bookmark7)

[Online Table 9. Multivariable model assessing predictors of 90-day mortality, using the](#_bookmark8) [quintiles values of elastic and resistive MP components at day 3 of MV](#_bookmark8) [simultaneously 19](#_bookmark8)

[Online Table 10. Multivariable model assessing predictors of 90-day mortality, using the](#_bookmark9) [quintiles values of the elastic static, elastic dynamic and resistive MP components at day 3 of](#_bookmark9) [MV simultaneously 20](#_bookmark9)

Online Table 11. Multivariable model assessing predictors of 90-day mortality, using the continuous values of PEEP at day 3 of MV 22

Online Table 12. Multivariable model assessing predictors of 90-day mortality, using the continuous values of the end-inspiratory plateau pressure at day 3 of MV 23

Online Figure 1. Receiver operating characteristic analysis and comparison of positive end-inspiratory pressure and elastic power at day 3 of MV in their capacity to predict 90-day mortality 24

Online Figure 2. Receiver operating characteristic analysis and comparison of positive end-expiratory pressure and elastic static power at day 3 of MV in their capacity to predict 90-day mortality 25

Online Figure 3. Acyclic graph 26

# Online Table 1. List of participating centers.

| **Site** |
| --- |
| Hospital Universitario Marqués de Valdecilla |
| Hospital Germans Trias i Pujol |
| Hospital Universitario de Torrejón |
| Hospital Universitari Joan XXIII de Tarragona |
| Hospital Universitario de Cruces |
| Hospital Universitario de Gran Canaria Doctor Negrín |
| Hospital General de Segovia |
| Hospital Universitari Son Espases |
| HM Hospitales Madrid |
| Hospital Virgen del Rocío |
| Hospital Virgen Macarena |
| Hospital General Universitario Gregorio Marañón |
| Hospital Universitario de Getafe |
| Hospital Universitario Ramón y Cajal |
| Hospital Universitario Río Hortega |
| Hospital Universitario Príncipe de Asturias |
| Hospital Punta de Europa-Algeciras |
| Hospital Universitario 12 de Octubre |
| Hospital Universitario Sant Joan d'Alacant |
| Hospital Universitario Lucus Augusti |
| Hospital Nuestra Señora de Gracia |
| Hospital Universitario de Móstoles |
| Hospital Universitari Bellvitge |
| Hospital Clinic Barcelona |
| Clínica Sagrada Familia |
| Hospital Universitario Vall d'Hebron |
| Hospital Clínico Universitario de Valladolid |
| Hospital de León |
| Hospital Universitari Arnau de Vilanova |
| Hospital San Pedro de Alcántara |
| Hospital Sagrat Cor |
| Hospital Infanta Leonor de Madrid |
| Hospital La Fe de Valencia |
| Hospital Universitari Mútua Terrassa |

| **Site** |
| --- |
| Hospital del Mar |
| Hospital de Mataró |
| Hospital Virgen De Valme |
| Complexo Hospitalario Universitario de Ourense |
| Hospital General Río Carrión |
| Hospital Universitario La Paz |
| Hospital Álvaro Cunqueiro |
| Hospital Universitario de Salamanca |
| Hospital de Tortosa Verge de la Cinta |
| Hospital Clínic Universitari de València |
| Hospital Son Llàtzer |
| Hospital de Santa Maria |
| Hospital Universitario de Jerez de la Frontera |
| Hospital Universitario San Agustín |
| Hospital Parc Taulí |
| Hospital Clínico Universitario de Santiago |

# Online Table 2. Quintiles of Mechanical Power and its components at MV start

| **Variables** | **n = 799** |
| --- | --- |
| MP |  |
| Q1 (<18 J/min) | 159 (19.9) |
| Q2 (≥18 - <21.7 J/min) | 160 (20) |
| Q3 (≥21.7 - <25.4 J/min) | 160 (20) |
| Q4 (≥25.4 - <30.6 J/min) | 160 (20) |
| Q5 (≥30.6 J/min) | 160 (20) |
| Elastic MP |  |
| Q1 (<13.2 J/min) | 159 (19.9) |
| Q2 (≥13.2 - <15.4 J/min) | 160 (20) |
| Q3 (≥15.4 - <17.6 J/min) | 159 (19.9) |
| Q4 (≥17.6 - <20.7 J/min) | 163 (20.4) |
| Q5 (≥20.7 J/min) | 158 (19.8) |
| Elastic, static MP |  |
| Q1 (<8.3 J/min) | 160 (20) |
| Q2 (≥8.3 - <10.1 J/min) | 160 (20) |
| Q3 (≥10.1 - <11.8 J/min) | 160 (20) |
| Q4 (≥11.8 - <14.1 J/min) | 159 (19.9) |
| Q5 (≥14.1 J/min) | 160 (20) |
| Elastic, dynamic MP |  |
| Q1 (<4 J/min) | 159 (19.9) |
| Q2 (≥4 - <4.9 J/min) | 160 (20) |
| Q3 (≥4.9- <6 J/min) | 160 (20) |
| Q4 (≥6 - <7.4 J/min) | 159 (19.9) |
| Q5 (≥7.4 J/min) | 161 (20) |
| Resistive MP |  |
| Q1 (<3.3 J/min) | 158 (19.8) |
| Q2 (≥3.3 - <5.5 J/min) | 162 (20.3) |
| Q3 (≥5.5 - <7.8 J/min) | 160 (20) |
| Q4 (≥7.8 - <11 J/min) | 159 (19.9) |
| Q5 (≥11 J/min) | 160 (20) |

Online Table 3. Effects of quantiles of Mechanical Power on outcomes, length of ICU stay, and duration of mechanical ventilation (day 3 of mechanical ventilation)

| **Total MP** | **Q1 (N = 61)** | **Q2 (N = 61)** | **Q3 (N = 62)** | **Q4 (N = 60)** | **Q5 (N = 62)** | **p-value** |
| --- | --- | --- | --- | --- | --- | --- |
| 90-day mortality^a^ | 15 (27.3) | 10 (18.9) | 15 (28.8) | 20 (42.6) | 23 (38.3) | 0.074 |
| 30-day mortality^b^ | 9 (15.5) | 3 (5.2) | 11 (19.3) | 17 (31.5)^c^ | 20 (32.3)^c^ | **0.001** |
| Length of ICU stay, days^d^ | 21 (15; 38) | 25 (13; 42) | 27 (16; 45) | 30 (14; 45) | 31 (16; 43) | 0.677 |
| Invasive MV length, days^d^ | 15 (12; 27) | 17 (10; 32) | 20 (13; 37) | 26 (11; 36) | 24 (12; 39) | 0.727 |
| **Elastic MP** | **Q1 (N = 60)** | **Q2 (N = 62)** | **Q3 (N = 61)** | **Q4 (N = 62)** | **Q5 (N = 61)** | **p-value** |
| 90-day mortality^a^ | 11 (22.4) | 12 (22.2) | 14 (29.2) | 19 (34.5) | 27 (44.3) | 0.061 |
| 30-day mortality^b^ | 7 (12.7) | 7 (11.9) | 7 (12.5) | 15 (25.9) | 24 (39.3)^cef^ | **<0.001** |
| Length of ICU stay, days^d^ | 19 (14; 33) | 21 (11; 47) | 30 (14; 42) | 34 (18; 49) | 30 (18; 43) | 0.062 |
| Invasive MV length, days^d^ | 15 (10; 27) | 14 (10; 30) | 26 (11; 36) | 24 (13; 41) | 22 (14; 40) | 0.133 |
| **Elastic static MP** | **Q1 (N = 61)** | **Q2 (N = 62)** | **Q3 (N = 61)** | **Q4 (N = 61)** | **Q5 (N = 61)** | **p-value** |
| 90-day mortality^a^ | 15 (33.3) | 8 (14) | 20 (37.7) | 16 (29.6) | 24 (41.4)^cf^ | **0.018** |
| 30-day mortality^b^ | 8 (14.3) | 5 (8.6) | 14 (24.1) | 12 (21.1) | 21 (35)^c^ | **0.006** |
| Length of ICU stay, days^d^ | 22 (13; 40) | 22 (15; 39) | 30 (13; 47) | 28 (16; 38) | 33 (18; 45) | 0.232 |
| Invasive MV length, days^d^ | 15 (10; 32) | 16 (12; 33) | 20 (11; 36) | 20 (11; 30) | 28 (14; 41) | 0.174 |

| **Elastic, dynamic MP** | **Q1 (N = 61)** | **Q2 (N = 61)** | **Q3 (N = 62)** | **Q4 (N = 61)** | **Q5 (N = 61)** | **p-value** |
| --- | --- | --- | --- | --- | --- | --- |
| 90-day mortality^a^ | 10 (18.5) | 13 (25) | 17 (32.7) | 19 (35.8) | 24 (42.9) | 0.058 |
| 30-day mortality^b^ | 7 (11.9) | 8 (13.8) | 10 (17.9) | 16 (28.1) | 19 (32.2)^e^ | **0.023** |
| Length of ICU stay, days^d^ | 21 (14; 47) | 27 (16; 40) | 17 (13; 34) | 30 (16; 45) | 34 (23; 51)^f^ | **0.039** |
| Invasive MV length, days^d^ | 17 (12; 32) | 22 (13; 31) | 13 (9; 29) | 25 (11; 36) | 24 (16; 43) | 0.080 |
| **Resistive MP** | **Q1 (N = 62)** | **Q2 (N = 60)** | **Q3 (N = 62)** | **Q4 (N = 61)** | **Q5 (N = 61)** | **p-value** |
| 90-day mortality^a^ | 20 (33.3) | 13 (23.6) | 13 (24.5) | 17 (39.5) | 20 (35.7) | 0.328 |
| 30-day mortality^b^ | 12 (19.7) | 7 (12.3) | 9 (15.5) | 14 (25.5) | 18 (31) | 0.093 |
| Length of ICU stay, days^d^ | 29 (15; 37) | 23 (13; 45) | 23 (15; 42) | 30 (15; 45) | 29 (15; 42) | 0.700 |
| Invasive MV length, days^d^ | 24 (14; 36) | 15 (10; 37) | 19 (11; 33) | 25 (10; 35) | 20 (11; 36) | 0.520 |

Data are presented as median (IQR) or as numbers (%). Percentages calculated on non-missing data. p-values marked in bold indicate numbers that are statistically significant on the 95% confidence limit.

Abbreviations: ICU, intensive care unit; IQR, interquartile range; MP, mechanical power; MV, mechanical ventilation; Q1, MP first quintile; Q2, MP second quintile; Q3, MP third quintile; Q4, MP fourth quintile; Q5, MP fifth quintile.

^a^ Calculated only for patients with 90-day follow-up (n = 267).

^b^ Calculated only for patients with 30-day follow-up (n = 289).

^c^ p<0.05 for comparison with Q2 (Bonferroni correction).

^d^ Calculated only for survivors (n = 83).

^e^ p<0.05 for comparison with Q1 (Bonferroni correction).

^f^ p<0.05 for comparison with Q3 (Bonferroni correction).

# Online Table 4. Multivariable model assessing predictors of 90-day mortality, using the continuous values of MP at day 3 of MV.

| **Variables** | **HR (95% CI)** | **P-value** |
| --- | --- | --- |
| *MODEL A* | | |
| Age (+1 year)^a^ | 1.06 (1.03 to 1.09) | **<0.001** |
| Male sex | 1.89 (1.00 to 3.59) | 0.051 |
| Days from initial symptoms to ICU admission (+1 day)^a^ | 1.00 (0.95 to 1.06) | 0.918 |
| APACHE-II score at ICU admission (+1)^a^ | 1.00 (0.94 to 1.05) | 0.873 |
| PaO2/FiO2 ratio at day 3 of MV (+1)^a^ | 1.00 (0.99 to 1.00) | 0.122 |
| pH at day 3 of MV (+1)^a^ | <0.001 (<0.001 to 0.04) | **<0.001** |
| Compliance at day 3 of MV (+1 mL/cmH2O)^a^ | 0.97 (0.94 to 1.00) | **0.042** |
| Ventilatory ratio at day 3 of MV (+1)^a^ | 1.07 (0.55 to 2.07) | 0.840 |
| Prone position at day 3 of MV | 1.44 (0.78 to 2.64) | 0.243 |
| MP at day 3 of MV (+1 J/min)^a^ | 1.03 (0.98 to 1.07) | 0.246 |
| Corticosteroid treatment | 0.98 (0.43 to 2.24) | 0.957 |
| *MODEL B* | | |
| Age (+1 year)^a^ | 1.06 (1.03 to 1.10) | **<0.001** |
| Male sex | 1.62 (0.84 to 3.12) | 0.147 |
| Days from initial symptoms to ICU admission (+1 day)^a^ | 1.00 (0.95 to 1.05) | 0.882 |
| APACHE-II score at ICU admission (+1)^a^ | 0.99 (0.93 to 1.05) | 0.729 |
| PaO2/FiO2 ratio at day 3 of MV (+1)^a^ | 1.00 (0.99 to 1.00) | 0.138 |
| pH at day 3 of MV (+1)^a^ | 0.001 (<0.001 to 0.04) | **0.001** |
| Compliance at day 3 of MV (+1 mL/cmH2O)^a^ | 0.97 (0.94 to 1.00) | 0.063 |
| Ventilatory ratio at day 3 of MV (+1)^a^ | 0.93 (0.47 to 1.83) | 0.823 |
| Prone position at day 3 of MV | 1.26 (0.68 to 2.34) | 0.463 |
| Elastic MP at day 3 of MV (+1 J/min)^a^ | 1.11 (1.02 to 1.21) | **0.014** |
| Resistive MP at day 3 of MV (+1 J/min)^a^ | 0.98 (0.92 to 1.05) | 0.612 |
| Corticosteroid treatment | 0.93 (0.40 to 2.13) | 0.860 |
| *MODEL C* | | |
| Age (+1 year)^a^ | 1.07 (1.04 to 1.10) | **<0.001** |
| Male sex | 1.95 (0.96 to 3.97) | 0.064 |
| Days from initial symptoms to ICU admission (+1 day)^a^ | 1.01 (0.96 to 1.07) | 0.670 |

| **Variables** | **HR (95% CI)** | **P-value** |
| --- | --- | --- |
| APACHE-II score at ICU admission (+1)^a^ | 1.00 (0.94 to 1.05) | 0.889 |
| PaO2/FiO2 ratio at day 3 of MV (+1)^a^ | 1.00 (0.99 to 1.00) | 0.107 |
| pH at day 3 of MV (+1)^a^ | 0.001 (<0.001 to 0.05) | **0.001** |
| Compliance at day 3 of MV (+1 mL/cmH2O)^a^ | 0.95 (0.90 to 0.99) | **0.024** |
| Ventilatory ratio at day 3 of MV (+1)^a^ | 1.02 (0.50 to 2.05) | 0.965 |
| Prone position at day 3 of MV | 1.11 (0.58 to 2.11) | 0.755 |
| Elastic, Static MP at day 3 of MV (+1 J/min)^a^ | 1.17 (1.05 to 1.31) | **0.006** |
| Elastic, Dynamic MP at day 3 of MV (+1 J/min)^a^ | 0.92 (0.71 to 1.20) | 0.541 |
| Resistive MP at day 3 of MV (+1 J/min)^a^ | 1.00 (0.94 to 1.07) | 0.987 |
| Corticosteroid treatment | 0.88 (0.42 to 2.32) | 0.983 |

*MODEL A*: using the continuous values of the MP at day 3 of MV; *MODEL B*: using the continuous values of the two MP components simultaneously (elastic and resistive) at day 3 of MV; *MODEL C*: using the continuous values of the three MP components simultaneously (elastic static, elastic dynamic and resistive) at day 3 of MV.

Data are shown as estimated HRs (95% CIs) of the explanatory variables in the 90-day mortality group. The p- value is based on the null hypothesis that all HRs relating to an explanatory variable equal unity (no effect). p- values marked in bold indicate numbers that are statistically significant on the 95% confidence limit.

Abbreviations: APACHE, acute physiology and chronic health evaluation; CI, confidence interval; FiO2, fraction of inspired oxygen; HR, hazard ratio; MP, mechanical power; MV, mechanical ventilation; PaO2, partial pressure of arterial oxygen.

^a^ “+1” means a one-unit increase on the scale in the predictor variable.

# Online Table 5. Multivariable model assessing predictors of 90-day mortality, using the quintiles values of MP at MV start.

| **Variables** | **HR (95% CI)** | **p-value** |
| --- | --- | --- |
| Age (+1 year)^a^ | 1.05 (1.03 to 1.06) | **<0.001** |
| Male sex | 1.12 (0.80 to 1.57) | 0.517 |
| Days from initial symptoms to ICU admission (+1 day)^a^ | 0.99 (0.97 to 1.02) | 0.690 |
| APACHE-II score at ICU admission (+1)^a^ | 1.04 (1.01 to 1.07) | **0.011** |
| PaO2/FiO2 ratio at ICU admission (+1)^a^ | 1.00 (1.00 to 1.00) | 0.365 |
| pH at ICU admission (+1)^a^ | 0.26 (0.05 to 1.19) | 0.083 |
| Compliance at MV start (+1 mL/cmH2O)^a^ | 0.99 (0.98 to 1.01) | 0.321 |
| Ventilatory ratio at MV start (+1)^a^ | 0.86 (0.67 to 1.10) | 0.230 |
| Prone position at MV start | 1.05 (0.77 to 1.43) | 0.742 |
| MP at MV start |  | 0.598 |
| Q1 (<18.1 J/min) | 1.00 | - |
| Q2 (≥18.1 - <22.2 J/min) | 1.13 (0.72 to 1.78) | 0.581 |
| Q3 (≥22.2 - <25.8 J/min) | 1.04 (0.63 to 1.71) | 0.889 |
| Q4 (≥25.8 - <31.4 J/min) | 1.06 (0.64 to 1.75) | 0.829 |
| Q5 (≥31.4 J/min) | 1.43 (0.83 to 2.47) | 0.201 |
| Corticosteroid treatment | 0.89 (0.54 to 1.48) | 0.666 |

Data are shown as estimated HRs (95% CIs) of the explanatory variables in the 90-day mortality group. The p- value is based on the null hypothesis that all HRs relating to an explanatory variable equal unity (no effect). p- values marked in bold indicate numbers that are statistically significant on the 95% confidence limit.

Abbreviations: APACHE, acute physiology and chronic health evaluation; CI, confidence interval; FiO2, fraction of inspired oxygen; HR, hazard ratio; MP, mechanical power; MV, mechanical ventilation; PaO2, partial pressure of arterial oxygen.

^a^ “+1” means a one-unit increase on the scale in the predictor variable.

# Online Table 6. Multivariable model assessing predictors of 90-day mortality, using the quintiles values of elastic and resistive MP components at MV start simultaneously.

| **Variables** | **HR (95% CI)** | **p-value** |
| --- | --- | --- |
| Age (+1 year)^a^ | 1.05 (1.03 to 1.06) | **<0.001** |
| Male sex | 1.13 (0.00 to 1.60) | 0.499 |
| Days from initial symptoms to ICU admission (+1 day)^a^ | 1.00 (0.97 to 1.02) | 0.721 |
| APACHE-II score at ICU admission (+1)^a^ | 1.04 (1.01 to 1.07) | **0.012** |
| PaO2/FiO2 ratio at ICU admission (+1)^a^ | 1.00 (1.00 to 1.00) | 0.404 |
| pH at ICU admission (+1)^a^ | 0.25 (0.05 to 1.17) | 0.079 |
| Compliance at MV start (+1 mL/cmH2O)^a^ | 0.99 (0.98 to 1.01) | 0.304 |
| Ventilatory ratio at MV start (+1)^a^ | 0.85 (0.66 to 1.09) | 0.197 |
| Prone position at MV start | 1.04 (0.76 to 1.42) | 0.819 |
| Elastic MP at MV start |  | 0.538 |
| Q1 (<13.5 J/min) | 1.00 | - |
| Q2 (≥13.5 - <15.8 J/min) | 0.98 (0.63 to 1.55) | 0.945 |
| Q3 (≥15.8 - <18.1 J/min) | 0.82 (0.51 to 1.33) | 0.428 |
| Q4 (≥18.1 - <21.2 J/min) | 0.94 (0.58 to 1.54) | 0.810 |
| Q5 (≥21.2 J/min) | 1.23 (0.72 to 2.10) | 0.451 |
| Resistive MP at MV start |  | 0.784 |
| Q1 (<3.3 J/min) | 1.00 | - |
| Q2 (≥3.3 - <5.5 J/min) | 1.13 (0.72 to 1.79) | 0.588 |
| Q3 (≥5.5 - <7.8 J/min) | 1.17 (0.72 to 1.90) | 0.536 |
| Q4 (≥7.8 - <11 J/min) | 1.01 (0.59 to 1.75) | 0.958 |
| Q5 (≥11 J/min) | 1.31 (0.75 to 2.31) | 0.346 |
| Corticosteroid treatment | 0.90 (0.54 to 1.49) | 0.675 |

Data are shown as estimated HRs (95% CIs) of the explanatory variables in the 90-day mortality group. The p- value is based on the null hypothesis that all HRs relating to an explanatory variable equal unity (no effect). p- values marked in bold indicate numbers that are statistically significant on the 95% confidence limit.

Abbreviations: APACHE, acute physiology and chronic health evaluation; CI, confidence interval; FiO2, fraction of inspired oxygen; HR, hazard ratio; MP, mechanical power; MV, mechanical ventilation; PaO2, partial pressure of arterial oxygen.

^a^ “+1” means a one-unit increase on the scale in the predictor variable.

## Online Table 7. Multivariable model assessing predictors of 90-day mortality, using the quintiles values of elastic static, elastic dynamic and resistive MP components at MV start simultaneously.

| **Variables** | **HR (95% CI)** | **p-value** |
| --- | --- | --- |
| Age (+1 year)^a^ | 1.05 (1.03 to 1.07) | **<0.001** |
| Male sex | 1.23 (0.87 to 1.73) | 0.251 |
| Days from initial symptoms to ICU admission (+1 day)^a^ | 0.99 (0.97 to 1.02) | 0.694 |
| APACHE-II score at ICU admission (+1)^a^ | 1.04 (1.01 to 1.07) | **0.01** |
| PaO2/FiO2 ratio at ICU admission (+1)^a^ | 1.00 (1.00 to 1.00) | 0.380 |
| pH at ICU admission (+1)^a^ | 0.23 (0.05 to 1.12) | 0.069 |
| Compliance at MV start (+1 mL/cmH2O)^a^ | 0.98 (0.96 to 1.00) | **0.024** |
| Ventilatory ratio at MV start (+1)^a^ | 0.89 (0.69 to 1.14) | 0.347 |
| Prone position at MV start | 1.04 (0.76 to 1.42) | 0.809 |
| Elastic, Static MP at MV start |  | 0.087 |
| Q1 (<8.6 J/min) | 1.00 | - |
| Q2 (≥8.6 - <10.2 J/min) | 0.83 (0.52 to 1.32) | 0.429 |
| Q3 (≥10.2 - <12.1 J/min) | 0.83 (0.51 to 1.35) | 0.456 |
| Q4 (≥12.1 - <14.3 J/min) | 0.75 (0.45 to 1.26) | 0.280 |
| Q5 (≥14.3 J/min) | 1.33 (0.75 to 2.36) | 0.321 |
| Elastic, Dynamic MP at MV start |  | 0.092 |
| Q1 (<4 J/min) | 1.00 | - |
| Q2 (≥4 - <4.9 J/min) | 0.40 (0.24 to 0.68) | **0.001** |
| Q3 (≥4.9- <6 J/min) | 0.46 (0.26 to 0.81) | **0.007** |
| Q4 (≥6 - <7.4 J/min) | 0.55 (0.30 to 1.02) | 0.059 |
| Q5 (≥7.4 J/min) | 0.44 (0.21 to 0.90) | **0.024** |
| Resistive MP at MV start |  | 0.640 |
| Q1 (<3.3 J/min) | 1.00 | - |
| Q2 (≥3.3 - <5.5 J/min) | 1.11 (0.70 to 1.76) | 0.657 |
| Q3 (≥5.5 - <7.8 J/min) | 1.17 (0.72 to 1.91) | 0.522 |
| Q4 (≥7.8 - <11 J/min) | 1.09 (0.63 to 1.89) | 0.757 |
| Q5 (≥11 J/min) | 1.48 (0.83 to 2.64) | 0.181 |

| **Variables** | **HR (95% CI)** | **p-value** |
| --- | --- | --- |
| Corticosteroid treatment | 0.86 (0.51 to 1.44) | 0.561 |

Data are shown as estimated HRs (95% CIs) of the explanatory variables in the 90-day mortality group. The p- value is based on the null hypothesis that all HRs relating to an explanatory variable equal unity (no effect). p- values marked in bold indicate numbers that are statistically significant on the 95% confidence limit.

Abbreviations: APACHE, acute physiology and chronic health evaluation; CI, confidence interval; FiO2, fraction of inspired oxygen; HR, hazard ratio; MP, mechanical power; MV, mechanical ventilation; PaO2, partial pressure of arterial oxygen.

^a^ “+1” means a one-unit increase on the scale in the predictor variable.

# Online Table 8. Multivariable model assessing predictors of 90-day mortality, using the quintiles values of the MP at day 3 of MV.

| **Variables** | **HR (95% CI)** | **p-value** |
| --- | --- | --- |
| Age (+1 year)^a^ | 1.06 (1.03 to 1.09) | **<0.001** |
| Male sex | 1.91 (1.02 to 3.60) | **0.044** |
| Days from initial symptoms to ICU admission (+1 day)^a^ | 1.00 (0.95 to 1.05) | 0.968 |
| APACHE-II score at ICU admission (+1)^a^ | 1.00 (0.95 to 1.06) | 0.930 |
| PaO2/FiO2 ratio at day 3 of MV (+1)^a^ | 1.00 (0.99 to 1.00) | 0.109 |
| pH at day 3 of MV (+1)^a^ | 0.001 (<0.001 to 0.06) | **0.001** |
| Compliance at day 3 of MV (+1 mL/cmH2O)^a^ | 0.97 (0.94 to 1.00) | **0.045** |
| Ventilatory ratio at day 3 of MV (+1)^a^ | 1.29 (0.85 to 1.97) | 0.236 |
| Prone position at day 3 of MV | 1.34 (0.73 to 2.48) | 0.348 |
| MP at day 3 of MV |  | 0.532 |
| Q1 (<17.6 J/min) | 1.00 | - |
| Q2 (≥17.6 - <21.1 J/min) | 0.72 (0.28 to 1.82) | 0.488 |
| Q3 (≥21.1 - <25.5 J/min) | 1.17 (0.48 to 2.89) | 0.728 |
| Q4 (≥25.5 - <32.1 J/min) | 1.04 (0.37 to 2.97) | 0.938 |
| Q5 (≥32.1 J/min) | 1.63 (0.55 to 4.82) | 0.374 |
| Corticosteroid treatment | 0.93 (0.40 to 2.16) | 0.861 |

Data are shown as estimated HRs (95% CIs) of the explanatory variables in the 90-day mortality group. The p- value is based on the null hypothesis that all HRs relating to an explanatory variable equal unity (no effect). p- values marked in bold indicate numbers that are statistically significant on the 95% confidence limit. Calculated only for patients with values of MP at day 3 of MV (n = 306).

Abbreviations: APACHE, acute physiology and chronic health evaluation; CI, confidence interval; FiO2, fraction of inspired oxygen; HR, hazard ratio; MP, mechanical power; MV, mechanical ventilation; PaO2, partial pressure of arterial oxygen.

^a^ “+1” means a one-unit increase on the scale in the predictor variable.

# Online Table 9. Multivariable model assessing predictors of 90-day mortality, using the quintiles values of elastic and resistive MP components at day 3 of MV simultaneously.

| **Variables** | **HR (95% CI)** | **p-value** |
| --- | --- | --- |
| Age (+1 year)^a^ | 1.06 (1.03 to 1.09) | <0.001 |
| Male sex | 1.60 (0.82 to 3.12) | 0.166 |
| Days from initial symptoms to ICU admission (+1 day)^a^ | 1.00 (0.95 to 1.05) | 0.968 |
| APACHE-II score at ICU admission (+1)^a^ | 1.02 (0.95 to 1.08) | 0.618 |
| PaO2/FiO2 ratio at day 3 of MV (+1)^a^ | 1.00 (0.99 to 1.00) | 0.114 |
| pH at day 3 of MV (+1)^a^ | <0.001 (<0.001 to 0.03) | **0.001** |
| Compliance at day 3 of MV (+1 mL/cmH2O)^a^ | 0.98 (0.95 to 1.01) | 0.258 |
| Ventilatory ratio at day 3 of MV (+1)^a^ | 1.15 (0.59 to 2.24) | 0.676 |
| Prone position at day 3 of MV | 1.33 (0.72 to 2.47) | 0.360 |
| Elastic Power at day 3 of MV |  | 0.227 |
| Q1 (<13.1 J/min) | 1.00 | - |
| Q2 (≥13.1 - <15.2 J/min) | 1.01 (0.39 to 2.61) | 0.987 |
| Q3 (≥15.2 - <17.5 J/min) | 1.50 (0.58 to 3.87) | 0.407 |
| Q4 (≥17.5 - <20.8 J/min) | 1.91 (0.72 to 5.06) | 0.191 |
| Q5 (≥20.8 J/min) | 3.31 (1.11 to 9.94) | 0.032 |
| Resistive Power at day 3 of MV |  | 0.402 |
| Q1 (<3.1 J/min) | 1.00 | - |
| Q2 (≥3.1 - <5.4 J/min) | 0.76 (0.32 to 1.80) | 0.533 |
| Q3 (≥5.4 - <7.9 J/min) | 0.52 (0.19 to 1.40) | 0.196 |
| Q4 (≥7.9 - <12.4 J/min) | 0.32 (0.10 to 1.04) | 0.057 |
| Q5 (≥12.4 J/min) | 0.53 (0.17 to 1.65) | 0.272 |
| Corticosteroid treatment | 0.88 (0.36 to 2.13) | 0.778 |

Data are shown as estimated HRs (95% CIs) of the explanatory variables in the 90-day mortality group. The p- value is based on the null hypothesis that all HRs relating to an explanatory variable equal unity (no effect). p- values marked in bold indicate numbers that are statistically significant on the 95% confidence limit. Calculated only for patients with values of MP at day 3 of MV (n = 306).

Abbreviations: APACHE, acute physiology and chronic health evaluation; CI, confidence interval; FiO2, fraction of inspired oxygen; HR, hazard ratio; MP, mechanical power; MV, mechanical ventilation; PaO2, partial pressure of arterial oxygen.

^a^ “+1” means a one-unit increase on the scale in the predictor variable.

# Online Table 10. Multivariable model assessing predictors of 90-day mortality, using the quintiles values of the elastic static, elastic dynamic and resistive MP components at day 3 of MV simultaneously.

| **Variables** | **HR (95% CI)** | **p-value** |
| --- | --- | --- |
| Age (+1 year)^a^ | 1.07 (1.04 to 1.10) | <0.001 |
| Male sex | 2.05 (1.00 to 4.22) | 0.050 |
| Days from initial symptoms to ICU admission (+1 day)^a^ | 1.00 (0.95 to 1.06) | 0.878 |
| APACHE-II score at ICU admission (+1)^a^ | 1.01 (0.95 to 1.08) | 0.753 |
| PaO2/FiO2 ratio at day 3 of MV (+1)^a^ | 1.00 (0.99 to 1.00) | 0.201 |
| pH at day 3 of MV (+1)^a^ | 0.001 (<0.001005 to 0.06) | 0.002 |
| Compliance at day 3 of MV (+1 mL/cmH2O)^a^ | 0.96 (0.92 to 1.01) | 0.123 |
| Ventilatory ratio at day 3 of MV (+1)^a^ | 1.27 (0.62 to 2.60) | 0.508 |
| Prone position at day 3 of MV | 1.30 (0.69 to 2.45) | 0.420 |
| Elastic, Static Power at day 3 of MV |  | 0.065 |
| Q1 (<8.6 J/min) | 1.00 | - |
| Q2 (≥8.6 - <9.9 J/min) | 0.59 (0.21 to 1.64) | 0.308 |
| Q3 (≥9.9 - <12 J/min) | 1.97 (0.76 to 5.11) | 0.163 |
| Q4 (≥12 - <14.1 J/min) | 1.34 (0.50 to 3.57) | 0.560 |
| Q5 (≥14.1 J/min) | 2.75 (0.88 to 8.60) | 0.082 |
| Elastic, Dynamic Power at day 3 of MV |  | 0.433 |
| Q1 (<3.9 J/min) | 1.00 | - |
| Q2 (≥3.9 - <4.8 J/min) | 1.98 (0.64 to 6.15) | 0.239 |
| Q3 (≥4.8- <5.8 J/min) | 1.65 (0.49 to 5.57) | 0.423 |
| Q4 (≥5.8 - <7 J/min) | 0.87 (0.23 to 3.38) | 0.845 |
| Q5 (≥7 J/min) | 1.44 (0.29 to 7.26) | 0.658 |
| Resistive Power at day 3 of MV |  | 0.527 |
| Q1 (<3.1 J/min) | 1.00 | - |
| Q2 (≥3.1 - <5.4 J/min) | 0.72 (0.31 to 1.71) | 0.463 |
| Q3 (≥5.4 - <7.9 J/min) | 0.61 (0.22 to 1.67) | 0.336 |
| Q4 (≥7.9 - <12.4 J/min) | 0.34 (0.10 to 1.15) | 0.082 |
| Q5 (≥12.4 J/min) | 0.53 (0.16 to 1.75) | 0.299 |

| **Variables** | **HR (95% CI)** | **p-value** |
| --- | --- | --- |
| Corticosteroid treatment | 0.68 (0.27 to 1.72) | 0.412 |

Data are shown as estimated HRs (95% CIs) of the explanatory variables in the 90-day mortality group. The p- value is based on the null hypothesis that all HRs relating to an explanatory variable equal unity (no effect). p- values marked in bold indicate numbers that are statistically significant on the 95% confidence limit. Calculated only for patients with values of MP at day 3 of MV (n = 306).

Abbreviations: APACHE, acute physiology and chronic health evaluation; CI, confidence interval; FiO2, fraction of inspired oxygen; HR, hazard ratio; MP, mechanical power; MV, mechanical ventilation; PaO2, partial pressure of arterial oxygen.

^a^ “+1” means a one-unit increase on the scale in the predictor variable.

# Online Table 11. Multivariable model assessing predictors of 90-day mortality, using the continuous values of PEEP at day 3 of MV.

| **Variables** | **HR (95% CI)** | **P-value** |
| --- | --- | --- |
| Age (+1 year)^a^ | 1.05 (1.02 to 1.08) | **0.001** |
| Male sex | 1.82 (1.00 to 3.29) | **0.048** |
| Days from initial symptoms to ICU admission (+1 day)^a^ | 0.99 (0.94 to 1.05) | 0.773 |
| APACHE-II score at ICU admission (+1)^a^ | 1.07 (1.01 to 1.13) | 0.022 |
| PaO2/FiO2 ratio at day 3 of MV (+1)^a^ | 1.00 (1.00 to 1.00) | 0.193 |
| pH at day 3 of MV (+1)^a^ | 0.80 (0.03 to 19.94) | 0.890 |
| Compliance at day 3 of MV (+1 mL/cmH2O)^a^ | 0.99 (0.97 to 1.01) | 0.544 |
| Ventilatory ratio at day 3 of MV (+1)^a^ | 0.79 (0.46 to 1.36) | 0.393 |
| Prone position at day 3 of MV | 1.03 (0.61 to 1.77) | 0.900 |
| PEEP at day 3 of VM (+1 cmH2O )^a^ | 1.13 (1.00 to 1.27) | 0.058 |
| Corticosteroid treatment | 0.98 (0.43 to 2.20) | 0.952 |

Abbreviations: MV indicates mechanical ventilation; HR, hazard ratio; CI, confidence interval; ICU, intensive care unit; APACHE, acute physiology and chronic health evaluation; PaO2, partial pressure of arterial oxygen; FiO2, fraction of inspired oxygen. Data are shown as estimated HRs (95% CIs) of the explanatory variables in the 90-day mortality group. The P-value is based on the null hypothesis that all HRs relating to an explanatory variable equal unity (no effect). P-values marked in bold indicate numbers that are statistically significant on the 95% confidence limit. Calculated only for patients with values of PEEP at day 3 of MV (n = 306).

Abbreviations: MV indicates mechanical ventilation; HR, hazard ratio; CI, confidence interval; ICU, intensive care unit; APACHE, acute physiology and chronic health evaluation; PaO2, partial pressure of arterial oxygen; PEEP, positive end-expiratory pressure; FiO2, fraction of inspired oxygen.

a “+1” means a one-unit increase on the scale in the predictor variable.

# Online Table 12. Multivariable model assessing predictors of 90-day mortality, using the continuous values of the end-inspiratory plateau pressure at day 3 of MV.

| **Variables** | **HR (95% CI)** | **P-value** |
| --- | --- | --- |
| Age (+1 year)^a^ | 1.07 (1.04 to 1.10) | **<0.001** |
| Male sex | 2.01 (1.09 to 3.73) | **0.026** |
| Days from initial symptoms to ICU admission (+1 day)^a^ | 1.00 (0.95 to 1.05) | 0.926 |
| APACHE-II score at ICU admission (+1)^a^ | 0.99 (0.94 to 1.05) | 0.807 |
| PaO2/FiO2 ratio at day 3 of MV (+1)^a^ | 1.00 (0.99 to 1.00) | 0.133 |
| pH at day 3 of MV (+1)^a^ | 0.001 (<0.00101 to 0.08) | **0.002** |
| Compliance at day 3 of MV (+1 mL/cmH2O)^a^ | 1.00 (0.97 to 1.04) | 0.972 |
| Ventilatory ratio at day 3 of MV (+1)^a^ | 1.20 (0.66 to 2.21) | 0.549 |
| Prone position at day 3 of MV | 1.25 (0.67 to 2.32) | 0.480 |
| End-inspiratory plateau pressure at day 3 of VM (+1 cmH2O )^a^ | 1.17 (1.04 to 1.33) | **0.010** |
| Corticosteroid treatment | 0.94 (0.41 to 2.18) | 0.891 |

Abbreviations: MV indicates mechanical ventilation; HR, hazard ratio; CI, confidence interval; ICU, intensive care unit; APACHE, acute physiology and chronic health evaluation; PaO2, partial pressure of arterial oxygen; FiO2, fraction of inspired oxygen. Data are shown as estimated HRs (95% CIs) of the explanatory variables in the 90-day mortality group. The P-value is based on the null hypothesis that all HRs relating to an explanatory variable equal unity (no effect). P-values marked in bold indicate numbers that are statistically significant on the 95% confidence limit. Calculated only for patients with values of End-inspiratory plateau pressure at day 3 of MV (n = 306).

Abbreviations: MV indicates mechanical ventilation; HR, hazard ratio; CI, confidence interval; ICU, intensive care unit; APACHE, acute physiology and chronic health evaluation; PaO2, partial pressure of arterial oxygen; FiO2, fraction of inspired oxygen.

a “+1” means a one-unit increase on the scale in the predictor variable.

# Online Figure 1. Receiver operating characteristic analysis and comparison of positive end-inspiratory pressure and elastic power at day 3 of MV in their capacity to predict 90-day mortality.

#
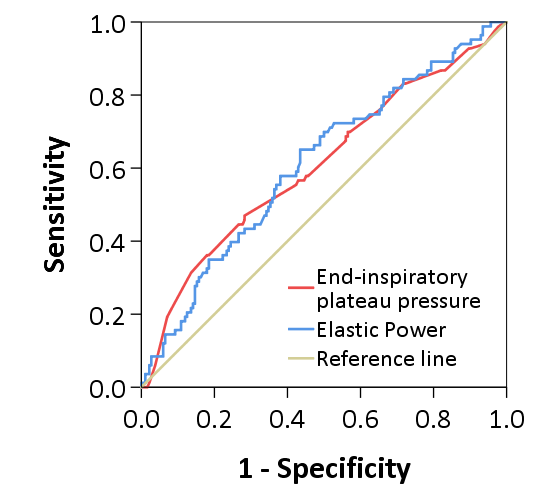


Abbreviations: MV indicates mechanical ventilation. AUC=0.61 (95% CI 0.53 to 0.68) for end-inspiratory plateau pressure and AUC=0.61 (95% CI 0.54 to 0.68) for elastic power. DeLong's test indicated a no significant difference between two AUCs (p=0.921).

# Online Figure 2. Receiver operating characteristic analysis and comparison of positive end-expiratory pressure and elastic static power at day 3 of MV in their capacity to predict 90-day mortality.


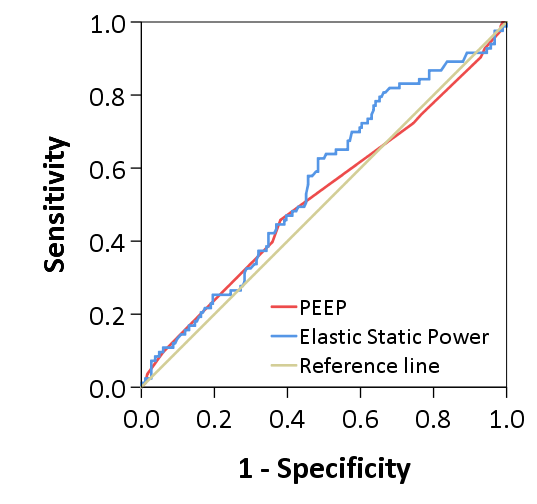


Abbreviations: MV indicates mechanical ventilation. AUC=0.52 (95% CI 0.44 to 0.60) for PEEP and AUC=0.56 (95% CI 0.48 to 0.63) for elastic stastic power. DeLong's test indicated a no significant difference between two AUCs (p=0.070).

# Online Figure 3. Acyclic graph.


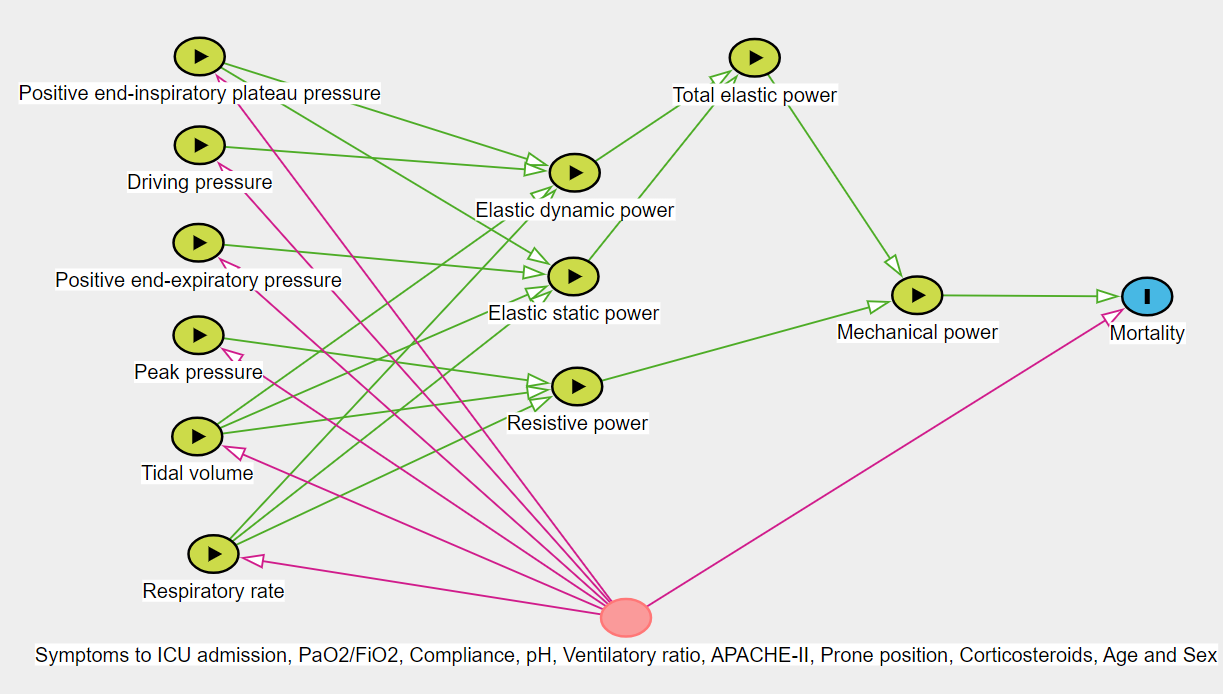

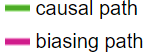

Supplement: Supplementary file 1 — Supplementary material 1. [file 13613_2025_1430_MOESM1_ESM.docx]
